# Design, Synthesis, Biological Evaluation, and *In Silico* Study of Tetrahydropyridines as Prospective Monoamine Oxidase Inhibitors

**DOI:** 10.1002/open.202400516

**Published:** 2025-06-12

**Authors:** Obaid ur Rehman Khan, Bilal Ahmad Khan, Syeda Shamila Hamdani, Saquib Jalil, Peter A. Sidhom, Khalid Elfaki Ibrahim, Tarad Abalkhail, Jamshed Iqbal, Hatem Tallima, Tamer Shoeib, Mahmoud A. A. Ibrahim

**Affiliations:** ^1^ Department of Chemistry The University of Azad Jammu and Kashmir Muzaffarabad 13100, Pakistan; ^2^ School of Packaging Michigan State University 448 Wilson Road East Lansing MI 48824 USA; ^3^ Center for Advanced Drug Research COMSATS University Islamabad Abbottabad Campus Abbottabad 22060 Pakistan; ^4^ Department of Pharmaceutical Chemistry, Faculty of Pharmacy Tanta University Tanta 31527 Egypt; ^5^ Department of Zoology, College of Science King Saud University P.O. Box 2455 Riyadh 11451 Saudi Arabia; ^6^ Department of Botany and Microbiology, College of Science King Saud University P.O. Box 2455 Riyadh 11451 Saudi Arabia; ^7^ Department of Chemistry The American University in Cairo New Cairo 11835 Egypt; ^8^ Computational Chemistry Laboratory, Chemistry Department, Faculty of Science Minia University Minia 61519 Egypt; ^9^ Department of Engineering, College of Engineering and Technology University of Technology and Applied Sciences Nizwa 611 Sultanate of Oman; ^10^ School of Health Sciences University of KwaZulu-Natal, Westville Campus Durban 4000 South Africa

**Keywords:** pyridine derivatives, monoamine oxidase inhibition, spectro-analytical techniques, molecular docking

## Abstract

The potentiality of monoamine oxidase (MAO) enzymes to break monoamine neurotransmitters makes them efficacious druggable targets. Molecules having MAO‐A inhibition characteristics are utilized as antidepressants while molecules with MAO‐B inhibition prospective are utilized to treat Parkinson's and Alzheimer's diseases. Herein, we have shown how the selective inhibition of both isozymes can be attained by varying the substitution of electron‐withdrawing and donating groups on the phenyl rings of tetrahydropyridines, i. e., ethyl 1,2,6‐triaryl‐4‐(arylamino)‐1,2,5,6‐tetrahydropyridine‐3‐carboxylate (**4 a**–**4 o**). The structures of these piperidines (**4 a**–**4 o**) were unambiguously established by different spectro‐analytical techniques, including ^1^H‐ and ^13^C‐NMR. Among the synthesized compounds, compounds **4 l** and **4 n** were identified as the most promising inhibitors of MAO‐A and MAO‐B, with IC_50_ values of 0.40±0.05 and 1.01±0.03 μM, respectively, compared with positive controls, namely clorgyline and l‐deprenyl, with IC_50_ values of 0.0045±0.0003 and 0.0196±0.001 μM, respectively. The binding interactions of the most potent derivatives within the binding pocket of the MAO‐A and MAO‐B enzymes were predicted by molecular docking studies. Binding mode analysis revealed the capacity of compounds **4 l** and **4 n** to exhibit a hydrogen bond with PHE177 of MAO‐A and GLN206 of MAO‐B, respectively.

## Graphical abstract

This work investigates the selective inhibition of MAO enzymes by varying the substitution of electron‐withdrawing and donating groups on the phenyl rings of tetrahydropyridines (**4 a**–**4 o**). Compounds **4 l** and **4 n** exhibit promising IC_50_ values of 0.40±0.05 and 1.01±0.03 μM against MAO‐A and MAO‐B, respectively. Molecular docking studies demonstrate the binding modes of **4l**  and **4 n** with MAO enzymes.

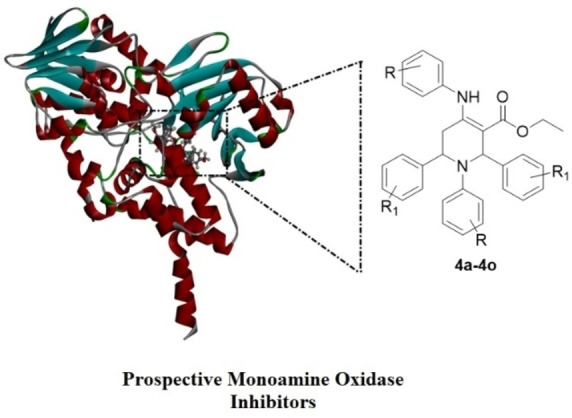

## 1 Introduction

Monoamine oxidase (MAO), an important flavoenzyme, is an intracellular enzyme present in the outer mitochondrial membrane of neuronal and non‐neuronal cells (glial) in the brain.[^1^, ^2^, ^3^] It is also present in many other cell types of peripheral organs.^[2]^ According to its biochemical and pharmacological functions, the MAO is categorized into two isoforms, namely MAO‐A and MAO‐B. These isoforms are encoded by separate genes on the X chromosome, and they possess 70 % sequence identity.^[4]^ Moreover, these isozymes are differentiated by their substrate and inhibitor selectivity because of the altered amino acid residues within the active sites and on tissue distribution.[^5^, ^6^, ^7^, ^8^, ^9^] In humans, MAO‐A is expressed in adipose tissue, lungs, placenta, and thyroid glands, whereas its expression in the brain is found to be very low.^[10]^ In comparison to MAO‐A, MAO‐B is mainly expressed in different parts of the central nervous system (CNS), such as basal ganglia, basal forebrain, thalamus, and brainstem, where it is responsible for up to ∼70 % of total brain activity.[^11^, ^12^, ^13^]

**Figure 1:**
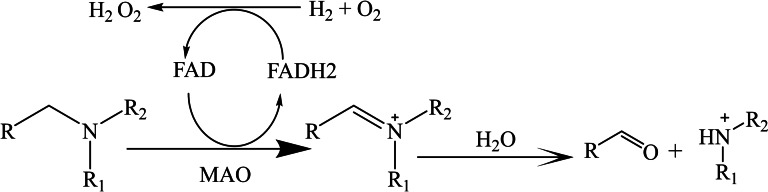
Reaction pathway of deamination of monoamine neurotransmitters by monoamine oxidase.^[20]^

Within the central and peripheral nervous systems, the physiological function of MAO‐A and MAO‐B involves the metabolism of exogenous biogenic amines and endogenous neurotransmitters, thus regulating the intracellular level of amine contents.[^14^, ^15^, ^16^, ^17^] On the other hand, in peripheral tissues, these isozymes are responsible for the oxidative deamination of amines, such as dopamine, tyramine, and tryptamine, to an intermediate amino product, which then generate their corresponding aldehydes along with ammonia and hydrogen peroxide as the side‐products (Figure 1).[^18^, ^19^]

The low levels of the neurotransmitters caused by aberrant expression of MAO‐A and MAO‐B in both the CNS and peripheral tissue are linked with various conditions, such as neurological and psychiatric disorders, cardiovascular disorders, MAO‐related oxidative stress, hypertension,^[21]^ diabetes, and aging.^[22]^ Therefore, the inhibition of the MAO isozymes with selective drugs has recently emerged as a pharmacological drug target for the treatment of psychiatric disorders, neurological disorders, and many others.[^23^, ^24^]

**Figure 2:**
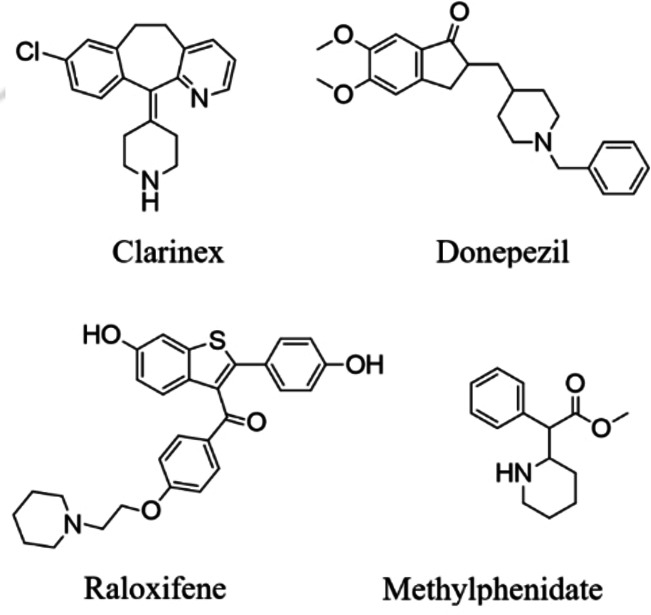
Examples of biologically active piperidine core‐containing compounds.

Piperidines are well‐known heterocyclic molecules with a variety of pharmacological applications, such as anti‐bacterial,^[25]^ anti‐fungal,^[26]^ elastase‐inhibitors,^[20]^ anti‐cancer,^[27]^ anti‐tuberculosis,^[28]^ anti‐analgesic, anti‐obesity,^[29]^ acetylcholine esterase inhibitor,^[30]^ antipsychotics,^[31]^ neuroprotective,^[32]^ antihypertensive,^[33]^ and antidepressant.[^34^, ^35^, ^36^, ^37^, ^38^] Piperidine is reported as a main chromophore for several drugs, like clarinex,^[39]^ donepezil,^[40]^ raloxifene,^[41]^ and methylphenidate[^42^, ^43^, ^44^, ^45^] (Figure 2). Because of the important role of piperidine derivatives as pharmacologically active structures in various curative medications, their synthesis has been widely explored in the literature.[^44^, ^46^, ^47^, ^48^] The tetrahydropyridine ring is found in many established medicinal compounds. A well‐established neurotoxin that induces symptoms similar to Parkinson's disease contains a tetrahydropyridine and is abbreviated as MPTP. Phenindamine, having a tetrahydropyridine ring, is an antihistamine and anticholinergic.^[49]^

Selectively oxidized piperidines, including pyridines, tetrahydropyridines, and dihydropyridines, constitute important scaffolds found in organic compounds with diverse biological activities, such as pyridines and dihydropyridines are found in natural products and have shown central nervous system activity.[^50^, ^51^, ^52^]

**Figure 3:**
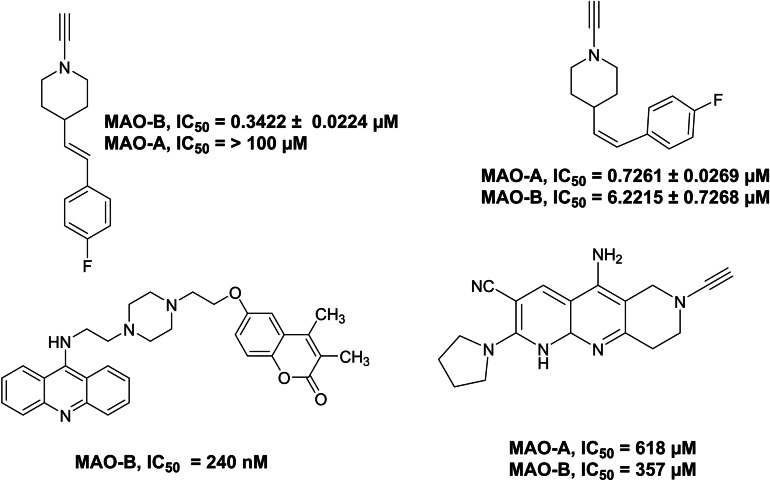
Examples of previously reported selective inhibitors of human MAO‐A and MAO‐B.

Moreover, the study of these drug‐like candidates based on structure‐activity relationships demonstrates the ongoing demand for tiny and medium‐sized molecules with a variety of substitution patterns.^[53]^ As a result, there is a continuing need to design new and effective processes for synthesizing this family of heterocyclic compounds. Recent studies have shown that geometric isomers of *cis*‐ and *trans*‐1‐propargyl‐4‐styrylpiperidines with different substitutions exhibit wide effects on MAO‐A and MAO‐B.^[54]^ According to the literature, *cis*‐isomers are strong inhibitors of human MAO‐A, while *trans*‐isomers are potent inhibitors of human MAO‐B (Figure 3).[^19^, ^55^] Clogyline and selegiline are the reported inhibitors of MAO‐A and MAO‐B, respectively. Clorgyline prevents the breakdown of neurotransmitters, such as serotonin and norepinephrine, which makes it an effective method for treating depression and anxiety disorders. In contrast, selegiline (also known as l‐deprenyl) inhibits MAO‐B selectively, resulting in a valuable treatment for Parkinson's disease by preventing the breakdown of dopamine, thus increasing its availability. Both compounds are essential tools for understanding and managing neurological conditions 1 and 2.

In continuation to our work on the synthesis and inhibition potential of tetrahydropyridines against monoamine oxidase^[56]^ and *α‐*amylase,^[57]^ herein, the synthesis of previously reported densely functional tetrahydropyridine‐based derivatives was achieved by the reaction of substituted aniline (**1**), ethyl acetoacetate (**2**), and substituted benzaldehyde (**3**).^[37]^

The synthesized derivatives (**4 a**–**4 o**) were first time evaluated as inhibitors of MAO‐A and MAO‐B. Most of the compounds exhibited promising MAO inhibition activity. Moreover, the synthesized compounds were further considered for docking computations to inspect their binding modes inside the binding sites of MAO targets.

## 2 Results and Discussion

### 2.1 Chemistry

**Figure 4:**
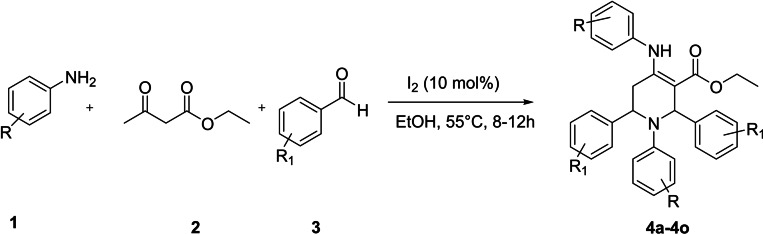
Synthetic scheme of tetrahydropyridines.

The synthetic way chosen to obtain the target tetrahydropyridine‐based derivatives is depicted in Figure 4. The compounds in Table 1 were freshly prepared following a reported procedure.[^20^, ^58^] In a straightforward and user‐friendly method to access tetrahydropyridines (**4 a**–**4 o**), substituted anilines were dissolved in the required amount of ethanol along with the addition of ethyl acetoacetate and a catalytic amount of molecular iodine and stirred at 55°C for 20 minutes. Substituted benzaldehydes were mixed with the components of the flask at the same temperature, and the reaction was monitored with thin‐layer chromatography (TLC) till the starting material disappeared. The precipitated crude product was obtained by cooling the flask to room temperature. The precipitates were washed with a minute amount of ethanol after filtration and recrystallized to obtain clean NMR spectroscopic data. It is worth mentioning that some of the synthesized compounds were previously reported.[^19^, ^55^, ^59^, ^60^, ^61^]

**Table 1:** Synthesis details of compounds′ structural parameters (**4 a**–**4 o**).

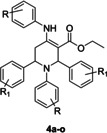			
Compound Name	R	R_1_	Yield%
**4 a**	H	H	80
**4 b**	4‐Cl	H	76
**4 c**	4‐Br	H	75
**4 d**	4‐OMe	H	88
**4 e**	4‐Me	H	87
**4 f**	H	4‐Cl	73
**4 g**	4‐Cl	4‐Cl	78
**4 h**	4‐Br	4‐Cl	75
**4 i**	4‐OMe	4‐Cl	80
**4 j**	4‐Me	4‐Cl	85
**4 k**	H	4‐NO_2_	75
**4 l**	4‐Cl	4‐NO_2_	75
**4 m**	4‐Br	4‐NO_2_	72
**4 n**	4‐OMe	4‐NO_2_	77
**4 o**	4‐Me	4‐NO_2_	78

The structures of the final products (**4 a**–**4 o**) were established on the basis of ^1^H‐ and ^13^C‐NMR spectroscopy and supported further by FT‐IR spectroscopy. In proton NMR spectroscopy, NH protons of compounds **4 a**–**4 o** were observed between *δ*H 10.15 and 10.66 ppm. The methane groups present in the cyclohexyl ring of compounds **4 a**–**4 o** were observed as a doublet, multiple, or broad signal between 4.99 and 5.41 ppm. Moreover, the structures of the desired compounds were confirmed by the appearance of a signal for CH_2_ protons in the piperidine ring; these two diastereotopic protons appear either as a separate signal or as a multiplet between *δ*H 4.25 and 4.57 ppm.

Methyl protons appeared as a triplet at *δ*H 1.39–1.53 ppm. The signals for the aromatic region appeared in the range of 6.12–8.20 ppm. The ^13^C‐NMR spectra of compounds **4 a**–**4 o** showed characteristic signals of the methylene group, which resonated between 59.36 and 60.64 ppm, while the methyl group appeared at 14.14 and 14.84 ppm. The presence of signals at 167.42–168.34 ppm for carbonyl groups confirms their synthesis. Because of the related coupling caused by the fluoro group that is directly linked to and next to the carbon, the trifluoromethyl signals (CF_3_) showed up as a pair of doublets. The presence of signals in the aromatic region between 108.44 and 159.71 ppm supported the confirmation of the final structure.

### 2.2 Biological Evaluation

The ability of all synthesized compounds **4 a**–**4 o** to inhibit the MAO‐A and MAO‐B enzymes was estimated. For this purpose, the clorgyline and l‐deprenyl were used as the positive controls for MAO‐A and MAO‐B, respectively. The inhibitory concentration (IC_50_) and the computed pIC_50_ values of all the tested compounds and standard inhibitors are shown in Table 2. All compounds showed promising inhibition of both MAOs in the lower micromolar range.

**Table 2:** Inhibitory concentration (IC_50_) and the computed pIC_50_ of the synthesized tetrahydropyridines (**4 a**–**4 o**) and standard compounds against MAO‐A and MAO‐B.

Compound Name	IC_50_±SEM (μM)^a^ or % inhibition^b^		pIC_50_	
	MAO‐A	MAO‐B	MAO‐A	MAO‐B
**4 a**	1.29±0.06^a^	1.70±0.09^a^	5.89	5.77
**4 b**	40 %^b^	1.54±0.13^a^	N/A	5.81
**4 c**	3.44±0.19^a^	22.3±2.71^a^	5.46	4.65
**4 d**	9.13±1.72^a^	8.89±0.13^a^	5.04	5.05
**4 e**	10.7±1.13^a^	8.03±0.86^a^	4.97	5.10
**4 f**	1.26±0.09^a^	42 %^b^	5.90	N/A
**4 g**	2.03±0.28^a^	2.07±0.01^a^	5.69	5.68
**4 h**	31 %^b^	19 %^b^	N/A	N/A
**4 i**	46 %^b^	44 %^b^	N/A	N/A
**4 j**	33 %^b^	44 %^b^	N/A	N/A
**4 k**	6.01±0.83^a^	8.47±0.14^a^	5.22	5.07
**4 l**	0.40±0.05^a^	23 %^b^	6.40	N/A
**4 m**	1.19±0.18^a^	46 %^b^	5.92	N/A
**4 n**	14.4±2.11^a^	1.01±0.03^a^	4.84	6.00
**4 o**	0.59±0.16^a^	2.67±0.09^a^	6.23	5.57
Clorgyline^c^	0.0045±0.0003^a^	61.3±1.13^a^		
L‐deprenyl^c^	67.25±1.020^a^	0.0196±0.001^a^		

[a] All the values are expressed as the mean±SEM of triplicate determinations. [b] Percent inhibition observed at 100 μM. [c] Standard drugs are used as a positive control.

### 2.3 Structure‐Activity Relationships (SAR)

The structure‐activity relationships (SAR) of all the synthesized compounds **4 a**–**4 o** were analyzed on both isoforms of the monoamine oxidase enzyme. The character of change over patterns of functional groups at the carboxylate ring was investigated to get selective inhibitors of monoamine oxidases.

**Figure 5:**
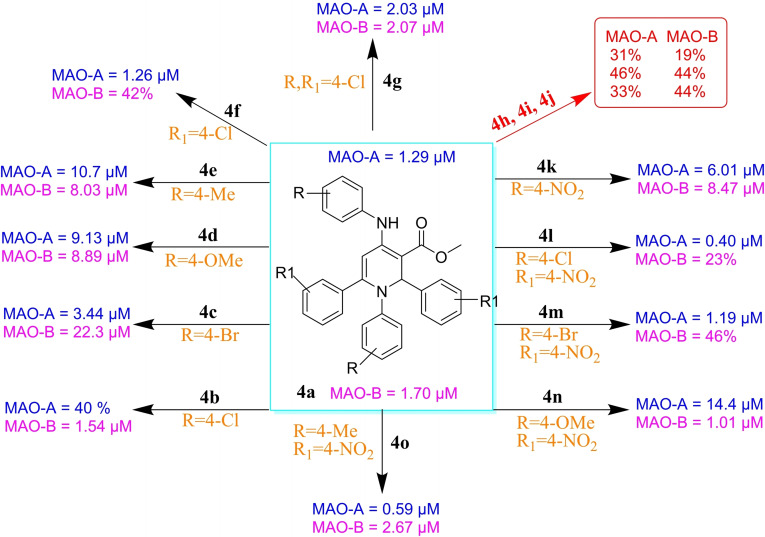
Graphical representation of SAR of tetrahydropyridines.

As shown in Table 1 and Figure 5, the basic structure of these compounds is composed of a tetrahydropyridine nucleus that is substituted with either R or R_1_ types of functional groups. Compound **4 a** can be designated as a lead compound that contains unsubstituted phenyl rings at the R and R_1_ positions. In order to draw a structure‐activity relationship, the remaining compounds were substituted with a variety of substituents at the R and R_1_ positions. Among compounds **4 a**–**4 e**, the R_1_ position was kept constant, and changes were introduced at the R position. Compound **4 a** had a good inhibitory profile and showed equipotent inhibition of both isoforms (i. e., MAO‐A and MAO‐B). Compound **4 b**, possessing 4‐Cl functionality at the R position, resulted in selective inhibition of MAO‐B with an IC_50_ value of 1.54±0.13 μM, corresponding to a pIC_50_ of approximately 5.81. Whereas compound **4 c** with a 4‐Br moiety at the R position displayed selectivity against MAO‐A (IC_50_±SEM=3.23±0.29 μM, pIC_50_≈5.49), as compared to MAO‐B (IC_50_±SEM=22.33±2.71 μM, pIC_50_≈4.65). On the other hand, incorporating the 4‐OCH_3_ functional group at the R position culminated in the dual inhibition of MAO‐A (IC_50_±SEM=9.13±1.72 μM, pIC_50_≈5.04) and MAO‐B (IC_50_±SEM=8.89±0.13 μM, pIC_50_≈5.05). Similarly, the inclusion of the 4‐OCH_3_ group at the R position made compound **4 e** a dual inhibitor of MAO‐A (IC_50_±SEM=10.7±1.13, pIC_50_≈4.97) and MAO‐B (IC_50_±SEM=8.03±0.86 μM, pIC_50_≈5.10). The comparable pIC_50_ values suggest that these compounds have nearly equal inhibitory effects on both enzymes.

Among compounds **4 f**–**4 j**, the 4‐Cl group was a constant at the R_1_ position, and changes were made at the R position to observe the effect of these modifications on the activity of enzymes. In this regard, the structure of compound **4 b** can be compared to that of compound **4 f**, where the 4‐Cl substitution was inserted at the R_1_ position instead of the R position. This alteration in the structure of compound **4 f** led to the selective inhibition of MAO‐A with an IC_50_ value of 1.26±0.09 μM, with a pIC_50_ value of approximately 5.90, thus emphasizing the influence of substituents′ positions on the selectivity of enzymes. In the case of compound **4 g**, where both the R and R_1_ positions were incorporating the same moiety (i. e., 4‐Cl), an equipotent effect on both MAO‐A and MAO‐B was observed. However, a reduced activity (less than 50 %) was displayed by compounds **4 h**, **4 i**, and **4 j**, indicating that 4‐Cl at the R_1_ position is best suited with 4‐Cl at the R position.

The compounds **4 k**–**4 o** were substituted with the 4‐NO_2_ functional group at the R_1_ position while incorporating different groups at the R position. The compounds **4 l** and **4 m** exhibited good inhibition against MAO‐A and MAO‐B enzymes. When the structure of these compounds was examined and compared with compound **4 k**, it was observed that they showed promising activity due to the presence of electronegative Cl and Br at the R position. However, the introduction of 4‐CH_3_ at R‐position in compound **4 o** resulted in improved activity towards MAO‐A rather than MAO‐B.

Conclusively, among different derivatives, the most promising inhibitor of MAO‐A was compound **4 l** with an IC_50_ value of 0.40±0.05 μM (pIC_50_ ≈6.40), while the potent inhibitor of MAO‐B was compound **4 n** with an IC_50_ value of 1.01±0.03 μM (pIC_50_ ≈5.99). Few compounds demonstrated promising MAO inhibition in the lower micromolar range. For instance, compound **4 b** showed selective inhibition of MAO‐B, and compounds **4 f**, **4 l**, and **4 m** showed selective inhibition of MAO‐A.

There was a strong selective effect of compound **4 b**, with a 4‐Cl group, on MAO‐B with a pIC_50_ of ≈5.81, while compound **4 c**, with a 4‐Br group, was more selective for MAO‐A (pIC_50_≈5.49). Compounds **4 d** and **4 e**, featuring a 4‐OCH_3_ group, displayed dual inhibition of both MAO‐A and MAO‐B enzymes, with similar pIC_50_ values of ≈5. Compound **4 l** emerged as the most potent MAO‐A inhibitor (pIC_50_≈6.40), while compound **4 n** was the most effective against MAO‐B (pIC_50_≈5.99). A significant influence was exerted by the type and positioning of substitutes on enzyme selectivity and potency.

### 2.4 *In Silico* Drug Discovery

#### 2.4.1 Molecular Docking

**Figure 6:**
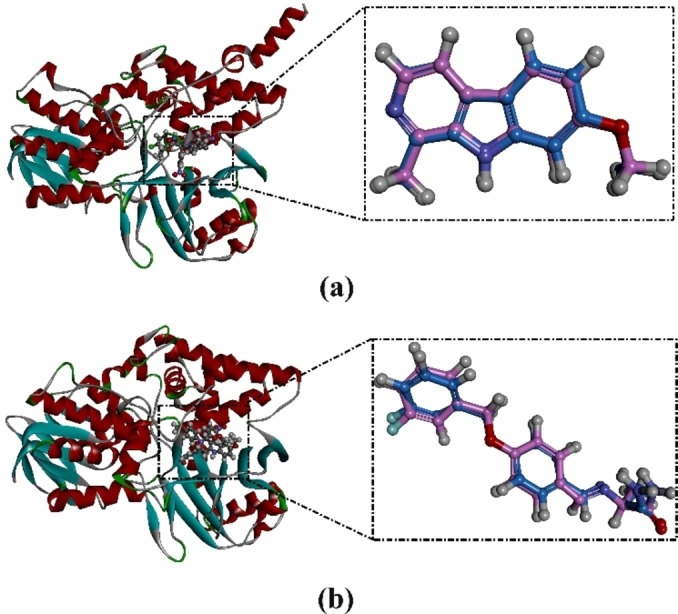
3D superimposition of the resolved experimental structure (pink) and the predicted docking pose (blue) of harmine with MAO‐A and (b) safinamide with MAO‐B.

The performance of AutoDock4.2.6 software in predicting the binding mode of the co‐crystallized harmine and safinamide within the MAO‐A and MAO‐B binding sites, respectively, was initially evaluated based on the available experimental data. The docking poses of harmine and safinamide were compared to their native structures (PDB codes: 2Z5X and 2V5Z, respectively) (Figure 6). As shown in Figure 6, the predicted docking poses were similar to the experimental binding poses. The RMSD observed between the predicted and experimental binding poses of harmine towards MAO‐A and safinamide against MAO‐B was determined to be 0.23 and 0.27 Å, respectively. Such observed low RMSD (<2.0 Å) suggests that the utilized docking technique is reliable for predicting the native binding mode of ligands inside the active site of MAO‐A and MAO‐B enzymes.

**Figure 7:**
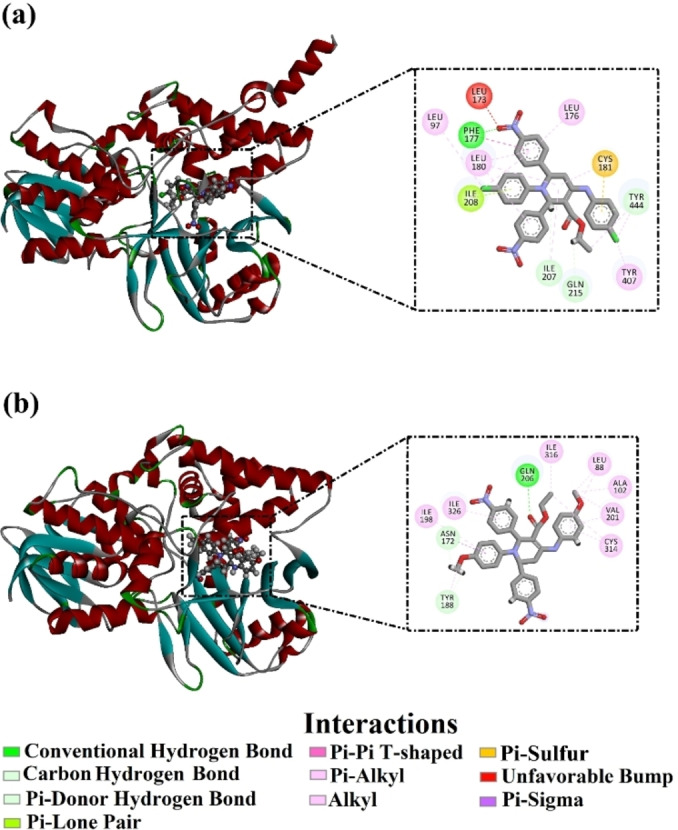
2D representations of compounds (a) **4 l** within the binding pocket of MAO‐A and (b) **4 n** within the binding pocket of MAO‐B.

The molecular docking technique was employed to anticipate the binding modes and docking scores of the synthesized tetrahydropyridines (**4 a**–**4 o**) against the MAO‐A and MAO‐B enzymes. Docking scores of these compounds against both MAOs were calculated, and the associated binding features were examined (Table S1). As shown in Table S1, a wide range of docking scores was noticed for the synthesized compounds with MAO‐A and MAO‐B, ranging from −6.2 to −10.2 and from −5.5 to −10.3 kcal/mol, respectively. Besides, the 2D binding modes for all investigated compounds against MAO‐A and MAO‐B are illustrated in Figures S1 and S2, respectively. Notably, the majority of the investigated compounds exhibited similar binding modes, establishing hydrogen bonds with key amino acid residues in the binding site of MAO‐A, including GLN215, PHE177, and CYS181 (Figure S1). However, all investigated compounds established a hydrogen bond with GLN206 in the binding site of MAO‐B (Figure S2). Compound **4 l** demonstrated the lowest docking score against the MAO‐A, with a value of −10.2 kcal/mol. Inspecting the binding mode of compound **4 l** revealed that 6‐(4‐nitrophenyl) of compound **4 l** played an important role in the inhibition of MAO‐A and formed a hydrogen bond with PHE177 (2.47 Å). As well, 6‐(4‐nitrophenyl) of compound **4 l** also exhibited π‐π stacking interaction with PHE177 (Figure 7a). The tetrahydropyridine ring of compound **4 l** demonstrated π‐sulfur, π‐π stacking, and π‐lone pair interactions with CYS181, ILE208, and TYR335 (Figure 7a). The presence of chlorine at position 4 in ring 1‐(4‐chlorophenyl) formed an π‐sigma interaction with TYR444 (Figure 7a). On the other hand, compound **4 n** unveiled the lowest docking score against MAO‐B, with a value of −10.3 kcal/mol. The binding interaction of compound **4 n** was inspected within the binding pocket of MAO‐B. Precisely, ethyl acetate of compound **4 n** established a hydrogen bond with GLN206 of MAO‐B with a bond length of 1.62 Å (Figure 7b). Besides, the anisole ring of the compound displayed π‐sigma interaction with ASN172 (Figure 7b).

#### 2.4.2 Drug‐Likeness Features

Drug‐likeness is a qualitative metric used in drug design to assess physicochemical characteristics like oral bioavailability.^[62]^ The physicochemical features were predicted using the SwissADME web tool^[63]^ and are summarized in Table 3. The cell membrane permeability, indicated by the Mlog *P* value, was 4.0 for compound **4 l** and 2.5 for compound **4 n**, suggesting good membrane permeability. Additionally, their molecular weights (MW) were 633.5 and 624.6 g/mol for compounds **4 l** and **4 n**, respectively, indicating readily diffused, transferred, and absorbed. Another indicator of molecular absorption is the topological polar surface area (TPSA), computed as the surface sum of polar atoms involving nitrogen, oxygen, and attached hydrogens. Molecules with a TPSA over 140 Å^2^ generally have poor membrane permeability, while a TPSA below 90 Å^2^ is typically considered favorable. Compounds **4 l** and **4 n** demonstrated TPSA with values of 133.2 and 151.7 Å^2^, respectively, revealing moderate membrane permeability and oral bioavailability. The number of HBD (hydrogen bond donor) was 1 for both compounds **4 l** and **4 n**. Ultimately, compounds **4 l** and **4 n** unveiled HBA (hydrogen bond acceptor) with a value of 6 and 8, respectively.

**Table 3:** Predicted drug‐likeness features of the compounds **4 l** and **4 n**.

Compound Name	MW	TPSA	Mlog *P*	HBD	HBA
**4 l**	633.5	133.2	4.0	1	6
**4 n**	624.6	151.7	2.5	1	8

#### 2.4.3 ADMET Features

Understanding pharmacokinetics and toxicity characteristics provides valuable insights for early‐stage drug discovery.^[64]^ Caco2 permeability and human intestinal absorption (HIA) are crucial absorption factors in any drug development process.[^65^, ^66^] The inspected compounds showed excellent absorption, with HIA values of 100 % for both compounds **4 l** and **4 n** (Table 4). Both compounds also exhibited adequate skin permeability, with values of −2.7 (Table 4). Additionally, the investigated compounds demonstrated favorable Caco2 permeability, being less than 0.9 cm/s. A fundamental ADMET property is whether a compound acts as a P‐glycoprotein substrate or inhibitor. Compounds **4 l** and **4 n** were classified as both inhibitors and substrates of P‐glycoprotein (Table 4). Drug distribution was assessed through blood‐brain barrier (BBB) permeability, the volume of distribution (VDss), and CNS penetration. Notable distribution volumes were observed, with log BB values of −0.9 for compound **4 l** and −1.0 for compound **4 n**, suggesting good BBB permeability (Table 4). For VDss and CNS permeability, compounds **4 l** and **4 n** had log VDss values of −1.0 and −1.5 and log PS values of −1.5 and −2.1, respectively.

**Table 4:** The predicted ADMET features for compounds **4 l** and **4 n**.

ADMET Characteristics	**4 l**	**4 n**
**Absorption**		
Skin permeability (log Kp)	−2.7	−2.7
Caco2 permeability (log Papp, cm/s)	0.2	−0.5
Intestinal absorption (human) (%)	100	100
P‐glycoprotein I inhibitor (Yes/No)	Yes	Yes
P‐glycoprotein substrate (Yes/No)	Yes	Yes
P‐glycoprotein II inhibitor (Yes/No)	Yes	Yes
**Distribution**		
VDss (human) (log L/kg)	−1.0	−1.5
CNS permeability (log PS)	−1.5	−2.1
BBB permeability (log BB)	−0.9	−1.0
**Metabolism**		
CYP2C19 inhibitor (Yes/No)	Yes	Yes
CYP1A2 inhibitor (Yes/No)	No	No
CYP3A4 inhibitor (Yes/No)	Yes	Yes
CYP2D6 inhibitor (Yes/No)	No	No
**Excretion**		
Total Clearance (log mL/min/kg)	−0.2	0.3
**Toxicity**		
Skin Sensitization (Yes/No)	No	No

Regarding drug metabolism, both compounds **4 l** and **4 n** were predicted to be non‐inhibitors of CYP2D6 and CYP1A2 enzymes and inhibitors of CYP2C19 and CYP3A4 enzymes. The total drug clearance was −0.2 mL/min/kg for compound **4 l** and 0.3 mL/min/kg for compound **4 n**. Specifically, compound **4I** demonstrated lower clearance, indicating prolonged systemic circulation and potential accumulation, whereas compound **4 n** revealed higher clearance, suggesting a faster elimination rate and a shorter half‐life. Toxicity plays a crucial role in selecting suitable drugs. Both compounds **4 l** and **4 n** showed no skin sensitization. Based on the predicted ADMET properties, these results suggest that the investigated compounds could serve as potential inhibitors for MAO enzymes.

## Experimental Details

### General

All starting materials were purchased from Sigma Aldrich in analytical grade from local suppliers and employed directly without any prior purification. The Gallenkamp melting point apparatus (MP−D) was used to find out the uncorrected melting points of pure products in open capillaries. The products were characterized by IR spectroscopy using the Shimadzu FT‐IR spectrometer IR‐Prestige21 (Shimadzu Co., Columbia, MD, USA) equipped with an attenuated‐total‐reflection accessory (ATR, PIKE Technologies, Madison, WI, USA). The ^1^H‐ and ^13^C‐NMR spectra (Figure S3) were recorded in Bruker AV‐300 spectrometer at 300 MHz, employing TMS as the internal standard and chloroform‐d6 as a solvent. The progress of the reaction and purity of tetrahydropyridines (**4 a**–**4 o**) was monitored and established by TLC using pre‐coated plates with silica gel‐60 F254 purchased from Merck Germany.

### General Procedure for the Synthesis of Tetrahydropyridines (4 a–4 o)

A single‐neck round‐bottom flask equipped with a magnetic stirrer was charged with substituted aniline (**1**) (2.0 mmol), ethyl acetoacetate (**2**) (1.0 mmol), and iodine (10 mol%) in ethanol. The mixture was stirred for 30 minutes at room temperature, followed by the addition of benzaldehyde (**3**) (2.0 mmol). The reaction mixture was further kept on stirring at 55–60 °C for 8–12 hours. The resulting precipitates were filtered and washed with cold ethanol twice to get pure piperidines (**4 a**–**4 o**). The final products were characterized by spectroscopic analysis and are described below:

#### Ethyl 1,2,6‐triphenyl‐4‐(phenylamino)‐1,2,5,6‐tetrahydropyridine‐3‐carboxylate (4 a)

White solid; yield 80 %; R_f_: 0.56 (n‐Hexane : ethyl acetate; 9 : 1); mp.: 174–176 °C; FT‐IR (cm^−1^): 3246 (NH), 2980 (*sp* ^3^ CH), 1650 (C=O), 1578 (C=C), 1326 (C−O), 1171, 749. ^1^H‐NMR (300 MHz, CDCl_3_) *δ*ppm: 1.50 (t, *J*=15 Hz, 3H), 2.78–2.94 (m, 2H), 4.33–4.52 (m, 2H), 5.17 (d, *J*=3.6 Hz, 1H), 6.30–6.32 (m, 2H), 6.50–6.64 (m, 2H), 7.07–7.38 (m, 16H), 10.33 (s, 1H).^13^C‐NMR (75 MHz, CDCl_3_) *δ*ppm: 14.83, 33.63, 55.07, 58.24, 59.71, 98.17, 112.94, 116.14, 125.69, 125.82, 126.30, 126.39, 126.64, 127.16, 128.26, 128.65, 128.84, 128.91, 137.88, 142.75, 144.02, 144.02, 146.97, 156.10, 168.27.

#### Ethyl 1‐(4‐chlorophenyl)‐4‐(4‐chlorophenylamino)‐2,6‐diphenyl‐1,2,5,6‐tetrahydropyridine‐3‐carboxylate (4 b)

White solid; yield 76 %; R_f_: 0.43 (n‐Hexane : ethyl acetate; 9 : 1); mp.: 223–224 °C; FT‐IR (cm^−1^): 3241 (NH), 2972 (*sp* ^3^ CH), 1644 (C=O), 1583 (C=C), 1318 (C−O), 1127, 727. ^1^H‐NMR (300 MHz, CDCl_3_) *δ*ppm: 1.50 (t, *J*=15 Hz, 3H), 2.69–2.91 (m, 2H), 4.33–4.53 (m, 2H), 5.13 (d, *J*=6 Hz, 1H), 6.18–6.21 (m, 2H), 6.41–6.47 (m, 3H), 7.00–7.09 (m, 4H), 7.16–7.32 (m, 10H), 10.26 (s, 1H).^13^C‐NMR (75 MHz, CDCl_3_) *δ*ppm:14.78, 33.46, 55.26, 58.33, 59.93, 98.71, 114.03, 121.24, 126.30, 126.50, 126.56, 127.03, 127.47, 128.38, 128.73, 128.82, 129.01, 131.36, 136.42, 142.26, 143.27, 145.50, 155.41, 168.15.

#### Ethyl 1‐(4‐bromophenyl)‐4‐(4‐bromophenylamino)‐2,6‐diphenyl‐1,2,5,6‐tetrahydropyridine‐3‐carboxylate (4 c)

White solid; yield 75 %; R_f_: 0.50 (n‐Hexane : ethyl acetate; 9 : 1); mp.: 230–232 °C; FT‐IR (cm^−1^): 3234 (NH), 2972 (*sp* ^3^ CH), 1644 (C=O), 1579 (C=C), 1317 (C−O), 1179, 772. ^1^H‐NMR (300 MHz, CDCl_3_) *δ*ppm:1.50 (t, *J*=15 Hz, 3H), 2.70–2.92 (m, 2H), 4.33–4.53 (m, 2H), 5.12 (d, *J*=3 Hz, 1H), 6.12–6.15 (m, 2H), 6.40–6.43 (m, 3H), 7.13–7.32 (m, 15H), 10.26 (s, 1H). ^13^C‐NMR (75 MHz, CDCl_3_) *δ*ppm: 14.78, 33.45, 55.21, 58.30, 59.96, 98.81, 108.44, 114.58, 119.14, 126.29, 126.48, 126.59, 127.28, 127.50, 128.40, 128.84, 131.61, 131.99, 136.93, 142.15, 143.16, 145.90, 155.26, 168.14.

#### Ethyl 1‐(4‐methoxyphenyl)‐4‐(4‐methoxyphenylamino)‐2,6‐diphenyl‐1,2,5,6‐tetrahydropyridine‐3‐carboxylate (4 d)

White solid; yield 88 %; R_f_: 0.40 (n‐Hexane : ethyl acetate; 9 : 1); mp.: 189–191 °C; FT‐IR (cm^−1^): 3235 (NH), 2933 (*sp* ^3^ CH), 1646 (C=O), 1579 (C=C), 1308 (C−O), 1108, 751. ^1^H‐NMR (300 MHz, CDCl_3_) *δ*ppm:1.47 (t, *J*=15 Hz, 3H), 2.63–2.85 (m, 2H), 3.68 (s, 3H), 3.76 (s, 3H), 4.28–4.52 (m, 2H), 5.07 (d, J=3 Hz, 1H), 6.21 (d, *J*=3 Hz, 2H), 6.37–6.48 (m, 2H), 6.62–6.67 (m, 4H), 7.19–7.36 (m, 10H), 10.16 (s, 1H).^13^C‐NMR (75 MHz, CDCl_3_) *δ*ppm: 14.83, 33.61, 55.39, 55.64, 55.67, 58.29, 59.52, 97.22, 113.93, 114.06, 114.50, 126.19, 126.53, 126.79, 127.08, 127.87, 128.14, 128.61, 130.73, 141.58, 143.29, 144.37, 150.88, 156.85, 157.75, 168.34.

#### Ethyl 4‐(p–toluidino)‐2,6‐diphenyl‐1‐p–tolyl‐1,2,5,6‐tetrahydropyridine‐3‐carboxylate (4 e)

White solid; yield 87 %; R_f_: 0.44 (n‐Hexane : ethyl acetate; 9 : 1); mp.; 198–199 °C; FT‐IR (cm^−1^): 3238 (NH), 3023 (*sp* ^2^ CH), 1646 (C=O), 1591 (C=C), 1315 (C−O), 1108, 793. ^1^H‐NMR (300 MHz, CDCl_3_) *δ*ppm: 1.48 (t, *J*=15 Hz, 3H), 2.18 (s, 3H), 2.28 (s, 3H), 2.73–2.90 (m, 2H), 4.32–4.50 (m, 2H), 5.14 (d, *J*=3 Hz, 1H), 6.18 (d, *J*=6 Hz, 2H), 6.45–6.47 (m, 2H), 6.89–6.92 (m, 4H), 7.19–7.38 (m, 10H), 10.24 (s, 1H).^13^C‐NMR (75 MHz, CDCl_3_) *δ*ppm:14.84, 20.14, 20.90, 33.56, 55.18, 58.24, 59.57, 97.69, 112.90, 125.04, 125.94, 126.19, 126.44, 126.67, 127.04, 128.20, 128.61, 129.41, 129.45, 135.23, 135.54, 143.04, 144.38, 144.86, 156.45, 168.30.

#### Ethyl 2,6‐bis(4‐chlorophenyl)‐1‐phenyl‐4‐(phenylamino)‐1,2,5,6‐tetrahydropyridine‐3‐carboxylate (4 f)

White solid; yield 73 %; R_f_: 0.51 (n‐Hexane : ethyl acetate; 9 : 1); mp.; 237–239 °C; FT‐IR (cm^−1^): 3233 (NH), 2871 (*sp* ^3^ CH), 1649 (C=O), 1580 (C=C), 1325 (C−O), 1174, 746; ^1^H‐NMR (300 MHz, CDCl_3_) *δ*ppm: 1.49 (t, *J*=15 Hz, 3H), 2.81–2.91(m, 2H), 4.34–4.52 (m, 2H), 5.14 (br. s., 1H), 6.41–6.52 (m, 5H), 6.66–6.71 (m, 1H), 7.09–7.42 (m, 14H), 10.34 (s, 1H). ^13^C‐NMR (75 MHz, CDCl_3_) *δ*ppm: 14.83, 33.72, 54.73, 57.40, 59.91, 97.81, 112.99, 116.03, 116.76, 123.87, 125.70, 125.97, 127.81, 128.06, 128.43, 128.80, 128.94, 129.04, 129.09, 132.14, 132.88, 137.69, 140.96, 142.48, 146.52, 155.84, 168.00.

#### Ethyl 1,2,6‐tris(4‐chlorophenyl)‐4‐(4‐chlorophenylamino)‐1,2,5,6‐tetrahydropyridine‐3‐carboxylate (4 g)

White solid; yield 78 %; R_f_: 0.40 (n‐Hexane : ethyl acetate; 9 : 1); mp.; 203–204 °C; FT‐IR (cm^−1^): 3234 (NH), 2862 (*sp* ^3^ CH), 1647 (C=O), 1586 (C=C), 1368 (C−O), 1185, 797. ^1^H‐NMR (300 MHz, CDCl_3_) *δ*ppm:1.47 (t, *J*=15 Hz, 3H), 2.67–2.87 (m, 2H), 4.31–4.50 (m, 2H), 5.07 (d, *J*=3 Hz, 1H), 6.30–6.40 (m, 5H), 7.02–7.07 (m, 4H), 7.12–7.17 (m, 2H), 7.20–7.29 (m, 6H), 10.27 (s, 1H). ^13^C‐NMR (75 MHz, CDCl_3_) *δ*ppm: 14.76, 33.58, 54.90, 57.44, 60.11, 98.25, 114.10, 121.90, 126.86, 127.67, 127.89, 128.55, 128.90, 128.95, 129.19, 131.64, 132.43, 133.22, 136.18, 140.35, 141.68, 145.01, 155.13, 167.87.

#### Ethyl 1‐(4‐bromophenyl)‐4‐(4‐bromophenylamino)‐2,6‐bis(4‐chlorophenyl)‐1,2,5,6‐tetrahydropyridine‐3‐carboxylate (4 h)

White solid; yield 75 %; R_f_: 0.55 (n‐Hexane : ethyl acetate; 9 : 1); mp.; 190–194 °C; FT‐IR (cm^−1^): 3233 (NH), 2859 (*sp* ^3^ CH), 1645 (C=O), 1582 (C=C), 1367 (C−O), 1179, 796. ^1^H‐NMR (300 MHz, CDCl_3_) *δ*ppm: 1.46 (t, J=15 Hz, 3H), 2.71–2.87 (m, 2H), 4.31–4.49 (m, 2H), 5.06 (br. s., 1H), 6.29–6.36 (m, 5H), 7.05–7.07 (m, 2H), 7.15–7.33 (10H), 10.26 (s, 1H).^13^C‐NMR (75 MHz, CDCl_3_) *δ*ppm: 14.73, 33.58, 57.42, 60.12, 114.72, 119.40, 127.10, 127.69, 127.90, 128.56, 128.96, 129.47, 130.91, 131.78, 132.17, 132.50, 133.28, 136.71, 154.94, 167.83.

#### Ethyl 2,6‐bis(4‐chlorophenyl)‐1‐(4‐methoxyphenyl)‐4‐(4‐methoxyphenylamino)‐1,2,5,6‐tetrahydropyridine‐3‐carboxylate (4 i)

White solid; yield 80 %; R_f_: 0.42 (n‐Hexane : ethyl acetate; 9 : 1); mp.; 227–229 °C; FT‐IR (cm^−1^): 2997 (NH), 2830 (*sp* ^3^ CH), 1656 (C=O), 1597 (C=C), 1364 (C−O), 1174, 791. ^1^H‐NMR (300 MHz, CDCl_3_) *δ*ppm: 1.43 (t, *J*=15 Hz, 3H), 2.62–2.78 (m, 2H), 3.69 (s, 3H), 3.78 (s, 3H), 4.25–4.48 (m, 2H), 4.99 (br. s., 1H), 6.35–6.41 (m, 4H), 6.67–6.71 (m, 4H), 7.06–7.09 (m, 2H), 7.24–7.28 (m, 7H), 10.17 (s, 1H).^13^C‐NMR (75 MHz, CDCl_3_) *δ*ppm: 14.77, 33.67, 55.43, 55.61, 57.33, 59.67, 96.69, 114.10, 114.55, 127.73, 127.97, 128.26, 128.72, 130.49, 132.00, 132.72, 141.00, 141.42, 142.71, 151.42, 156.51, 157.93, 168.08.

#### Ethyl 4‐(p–toluidino)‐2,6‐bis(4‐chlorophenyl)‐1‐p–tolyl‐1,2,5,6‐tetrahydropyridine‐3‐carboxylate (4 j)

White solid; yield 85 %; R_f_: 0.58 (n‐Hexane : ethyl acetate; 9 : 1); mp.; 229–231 °C; FT‐IR (cm^−1^): 3247 (NH), 2976 (*sp* ^2^ CH), 2917 (*sp* ^3^ CH), 1652 (C=O), 1572 (C=C), 1324 (C−O), 1175, 789. ^1^H‐NMR (300 MHz, CDCl_3_) *δ*ppm: 1.46 (t, *J*=15 Hz, 3H), 2.20 (s, 3H), 2.31 (s, 3H), 2.71–2.84 (m, 2H), 4.31–4.48 (m, 2H), 5.08 (br. s., 1H), 6.30–6.40 (m, 5H), 6.90–7.10 (m, 6H), 7.25–7.28 (m, 6H), 10.24 (s, 1H).^13^C‐NMR (75 MHz, CDCl_3_) *δ*ppm:14.80, 20.13, 20.19, 33.62, 54.87, 57.36, 59.73, 97.25, 113.01, 125.83, 127.86, 128.08, 128.34, 128.73, 129.58, 132.01, 132.76, 135.02, 135.88, 141.22, 142.79, 144.37, 156.14, 168.01.

#### Ethyl 2,6‐bis(4‐nitrophenyl)‐1‐phenyl‐4‐(phenylamino)‐1,2,5,6‐tetrahydropyridine‐3‐carboxylate (4 k)

White solid; yield 75 %; R_f_: 0.30 (n‐Hexane : ethyl acetate; 9 : 1); mp.; 239–240 °C; FT‐IR (cm^−1^): 3237 (NH), 2971 (*sp* ^2^ CH), 1654 (C=O), 1591 (C=C), 1342 (C−O), 1175, 748. ^1^H‐NMR (300 MHz, CDCl_3_) *δ*ppm:1.50 (t, *J*=15 Hz, 3H), 2.89–2.90 (m, 2H), 4.35‐4.54 (m, 2H), 5.27‐5.30 (m, 1H), 6.41–6.50 (m, 5H), 6.72 (t, *J*=15 Hz, 1H), 7.09–7.20 (m, 5H), 7.28–7.33 (m, 2H), 7.52–7.55 (m, 2H), 8.14–8.20 (m, 4H), 10.34 (s, 1H). ^13^C‐NMR (75 MHz, CDCl_3_) *δ*ppm:14.78, 33.65, 57.42, 60.27, 96.73, 113.26, 123.64, 123.78, 123.96, 125.48, 126.39, 127.42, 127.50, 129.23, 129.38, 137.20, 145.56, 146.87, 147.35, 149.53, 151.42, 155.25, 167.59.

#### Ethyl 1‐(4‐chlorophenyl)‐4‐(4‐chlorophenylamino)‐2,6‐bis(4‐nitrophenyl)‐1,2,5,6‐tetrahydropyridine‐3‐carboxylate (4 l)

White solid; yield 75 %; R_f_: 0.30 (n‐Hexane : ethyl acetate; 9 : 1); mp.; 229–230 °C; IR (cm^−1^): 2978 (NH), 1649 (C=O), 1587 (C=C), 1346 (C−O), 1172, 735. ^1^H‐NMR (300 MHz, CDCl_3_) *δ*ppm: 1.50 (t, *J*=15 Hz, 3H), 2.87–2.90 (m, 2H), 4.34–4.57 (m, 2H), 5.24–5.26 (m, 1H), 6.28–6.42 (m, 5H), 7.17–7.20 (m, 2H), 7.48–7.51 (m, 2H), 8.15–8.20 (m, 4H), 10.31 (s, 1H). ^13^C‐NMR (75 MHz, CDCl_3_) δppm: 14.76, 33.54, 55.48, 57.37, 60.53, 97.52, 110.22, 114.71, 119.82, 123.90, 124.07, 126.79, 127.29, 132.14, 132.41, 136.25, 144.73, 147.00, 147.51, 148.88, 150.71, 154.47, 167.47.

#### Ethyl 1‐(4‐bromophenyl)‐4‐(4‐bromophenylamino)‐2,6‐bis(4‐nitrophenyl)‐1,2,5,6‐tetrahydropyridine‐3‐carboxylate (4 m)

White solid; yield 72 %; R_f_: 0.20 (n‐Hexane : ethyl acetate; 9 : 1); mp.; 2236–237 °C; FT‐IR (cm^−1^): 3234 (NH), 2970 (*sp* ^2^ CH), 1648 (C=O), 1582 (C=C), 1327 (C−O), 1175, 753. ^1^H‐NMR (300 MHz, CDCl_3_) *δ*ppm: 1.50 (t, *J*=15 Hz, 3H), 2.87–2.90 (m, 2H), 4.34–4.57 (m, 2H), 5.24–5.26 (m, 1H), 6.28–6.42 (m, 5H), 7.17–7.20 (m, 2H), 7.48–7.51 (m, 2H), 8.15–8.20 (m, 4H), 10.31 (s, 1H). ^13^C‐NMR (75 MHz, CDCl_3_) *δ*ppm:14.76, 33.54, 55.48, 57.37, 60.53, 97.52, 110.22, 114.71, 119.82, 123.90, 124.07, 126.79, 127.29, 132.14, 132.41, 136.25, 144.73, 147.00, 147.51, 148.88, 150.71, 154.47, 167.47.

#### Ethyl 1‐(4‐methoxyphenyl)‐4‐(4‐methoxyphenylamino)‐2,6‐bis(4‐nitrophenyl)‐1,2,5,6‐tetrahydropyridine‐3‐carboxylate (4 n)

White solid; yield 77 %; R_f_: 0.30 (n‐Hexane : ethyl acetate; 9 : 1); mp.; 214–215 °C; FT‐IR (cm^−1^): 3244 (NH), 2937 (*sp* ^2^ CH), 2840 (*sp* ^3^ CH), 1649 (C=O), 1578 (C=C), 1346 (C−O), 1174, 742. ^1^H‐NMR (300 MHz, CDCl_3_) *δ*ppm: 1.39 (t, *J*=15 Hz, 3H), 2.76–2.89 (m, 2H), 3.68 (s, 3H), 3.80 (s, 3H), 4.27–4.46 (m, 2H), 5.12–5.15 (m, 1H), 6.30–6.48 (m, 5H), 6.66–6.73 (m, 4H), 7.34–7.48 (m, 4H), 8.12–8.17 (m, 4H), 10.24 (s, 1H). ^13^C‐NMR (75 MHz, CDCl_3_) *δ*ppm: 14.73, 33.68, 55.46, 55.58, 57.38, 60.03, 114.32, 114.74, 115.35, 123.60, 123.90, 127.53, 127.65, 130.00, 146.79, 147.28, 155.90, 158.19, 167.69.

#### Ethyl 4‐(p–toluidino)‐2,6‐bis(4‐nitrophenyl)‐1‐p–tolyl‐1,2,5,6‐tetrahydropyridine‐3‐carboxylate (4 o)

White solid; yield 78 %; R_f_: 0.20 (n‐Hexane : ethyl acetate; 9 : 1); mp.; 184–186 °C; FT‐IR (cm^−1^): 3233 (NH), 2973 (*sp* ^2^ CH), 2858 (*sp* ^3^ CH), 1650 (C=O), 1594 (C=C), 1346 (C−O), 1177, 748. ^1^H‐NMR (300 MHz, CDCl_3_) δppm:1.41 (t, J=15 Hz, 3H), 2.19 (s, 1H), 2.34 (s, 1H), 2.80–2.97 (m, 2H), 4.29–4.45 (m, 2H), 5.20–5.22 (m, 1H), 6.39–6.43 (m, 5H), 6.90–7.01 (m, 4H), 7.36–7.50 (m, 4H), 8.12–8.18 (m, 4H), 10.31 (s, 1H). ^13^C‐NMR (75 MHz, CDCl_3_) *δ*ppm: 14.76, 20.16, 20.92, 33.60, 57.42, 60.12, 123.70, 123.93, 125.63, 127.59, 129.80, 129.87, 134.54, 136.41, 146.82, 147.30, 155.53, 167.61.

### MAO Activity

The assay was conducted as previously described in a white 96‐well plate.^[67]^ Prior to utilization, MAO‐A and MAO‐B enzymes were irreversibly inhibited with clorgyline and l‐deprenyl, respectively, at 25 °C for 15–20 min. The assay volume for MAO‐A was 200 μL, involving 145 μL of buffer (NaH_2_PO_4_, pH 7.4), 20 μL (26 μg end concentration), and 10 μL of test compound (100 μM end concentration) of freshly prepared enzyme. The range of compounds used in the study started at 1 mM for the initial screening to determine the percentage of inhibition. For IC_50_ determination, eight serial dilutions were used: 1 mM, 0.3 mM, 0.1 mM, 0.003 mM, 0.001 mM, 0.0003 mM, 0.0001 mM, and 0.00003 mM. For 10 minutes, the reaction mixture was incubated at 37 °C. Tyramine, a substrate with a final concentration of 0.30 mM, was added to each well after 15 minutes, followed by 10 μL of Amplex red, a fluorogenic substrate. The FLUOstar Omega fluorescence plate reader (BMG Labtech GmbH, Ortenberg, Germany) was used to detect the change in fluorescence. Further research was conducted on the substances that inhibited MAO‐A or MAO‐B by more than 50 %, and IC_50_ values were computed using the PRISM 5.0 (GraphPad, San Diego, California, USA) software.

### *In Silico* Computations

#### Protein Preparation

The crystal structures of MAO‐A (PDB ID: 2Z5X^[68]^) and MAO‐B (PDB ID: 2V5Z^[5]^) were downloaded and utilized for docking computations. The protein was prepared by assigning bonds in the proper order, reorienting groups that were confused, and eliminating co‐crystallized water molecules and inhibitors. Using the H++ web server, the protonation states of the residues were determined, and the missing hydrogens were added.^[69]^

#### Ligand Preparation

The 3D structures of the synthesized compounds were generated utilizing Omega2 software with a maximum of 200 conformers generated within a 10 kcal/mol energy window.^[70]^ Using the MMFF94S force field integrated into the SZYBKI software, the created structures were energetically minimized.[^71^, ^72^]

#### Molecular Docking

Docking computations were executed using AutoDock4.2.6 software.^[73]^ Using MGL tools, the pdbqt file for the MAO‐A and MAO‐B was created.^[74]^ All docking parameters were left at their default settings, with the exception of the *eval* (maximum number of energy evaluations) and *GA* (number of genetic algorithms executed) values. The *GA* and *eval* were 250 and 25,000,000, respectively. The grid box dimensions were 50 Å × 50 Å × 50 Å. The grid maps with a spacing of 0.375 Å were made using the AutoGrid4.2.6 program. The coordinates of MAO‐A grid center were X=−39.434, Y=−26.373, and Z=−14.903. The MAO‐B grid had coordinates of X=−41.463, Y=−19.932, and Z=−20.27 Å. The built‐in clustering analysis was employed to categorize the predicted docking poses for every compound, applying an RMSD tolerance of 1.0 Å. The Discovery Studio module of the Biovia software was used to visualize all molecular interactions.^[75]^

#### Drug‐Likeness Features

The drug‐likeness properties of the most potent compounds were assessed using the SwissADME server.^[63]^ According to Lipinski's rule, five key factors were evaluated: molecular weight (MW≤500 g/mol), hydrogen bond donors (HBD≤5), log *P* (Mlog *P*≤5) indicating good oral and intestinal absorption, topological polar surface area (TPSA≤140 Å^2^) signifying excellent oral absorption or membrane permeability, and hydrogen bond acceptors (HBA≤10). Meeting these criteria suggests that the investigated compounds in question are orally bioavailable.

#### ADMET Features

The online pkCSM tool was used to predict the ADMET properties of the most potent compounds.^[76]^ Absorption (A) includes skin permeability, Caco‐2 permeability, human intestinal absorption (HIA), skin permeability, and whether the compound is a P‐glycoprotein substrate or inhibitor. Distribution (D) covers aspects, such as fraction unbound, central nervous system (CNS) permeability, blood‐brain barrier (BBB) permeability, and steady‐state volume of distribution (VDss). Metabolism (M) relies on factors like cytochrome P450 inhibitors. Excretion (E) is assessed by total clearance via inhibitors, while toxicity (T) is predicted based on skin sensitization.

## 3 Conclusions

In the current study, we have identified a number of compounds of tetrahydropyridine derivatives (**4 a**–**4 o**) possessing potent MAO inhibitory activities. Among the investigated compounds, compound **4 l** demonstrated promising activity with an IC_50_ value of 0.40±0.05 μM against MAO‐A. For MAO‐B, compound **4 n** showed an IC_50_ value of 1.01±0.03 μM. The binding interactions between the potent compounds and the MAO enzyme were also determined, and it was found that the identified compounds showed strong interactions with the key amino acid residues inside the binding pockets. As well, the physicochemical features demonstrated good oral bioavailability for the investigated compounds. The investigated compounds also revealed promising ADMET characteristics. The current findings shine new light on the potentiality of tetrahydropyridines as MAO inhibitors.

## Supporting Information

2D representation of the predicted binding mode for the synthesized tetrahydropyridines (**4 a**–**4 o**) against MAO‐A enzyme (Figure S1). 2D representation of the predicted binding mode for the synthesized tetrahydropyridines (**4 a**–**4 o**) against MAO‐B enzyme (Figure S2). ^1^H‐NMR and ^13^C‐NMR spectra of compounds **4 a**–**4 o** (Figure S3). The predicted docking scores and the binding features of the synthesized tetrahydropyridines (**4 a**–**4 o**) against MAO‐A and MAO‐B enzymes (Table S1). IC_50_ graphs of compounds **4 a**–**4 o** against MAO‐A and MAO‐B enzymes (Table S2).

## Conflict of Interests

The authors declare no conflict of interest.

## Supporting information

As a service to our authors and readers, this journal provides supporting information supplied by the authors. Such materials are peer reviewed and may be re‐organized for online delivery, but are not copy‐edited or typeset. Technical support issues arising from supporting information (other than missing files) should be addressed to the authors.

Supporting Information

## Data Availability

The data that support the findings of this study are available in the supplementary material of this article.
