# Supplementary material for: Design, Synthesis, Biological Evaluation, and In Silico Study of Tetrahydropyridines as Prospective Monoamine Oxidase Inhibitors
Source: ChemistryOpen. 2025 Jun 12;14(10):e202400516. doi: 10.1002/open.202400516 (PMC12518037; doi:10.1002/open.202400516)
Supplement: Supplementary file 1 — Supporting Information [file OPEN-14-e202400516-s001.pdf]

ChemistryOpen

Supporting Information

Design, Synthesis, Biological Evaluation, and *In Silico* Study of Tetrahydropyridines as Prospective Monoamine Oxidase Inhibitors

Obaid ur Rehman Khan, Bilal Ahmad Khan,* Syeda Shamila Hamdani, Saquib Jalil, Peter A. Sidhom, Khalid Elfaki Ibrahim, Tarad Abalkhail, Jamshed Iqbal, Hatem Tallima, Tamer Shoeib, and Mahmoud A. A. Ibrahim*

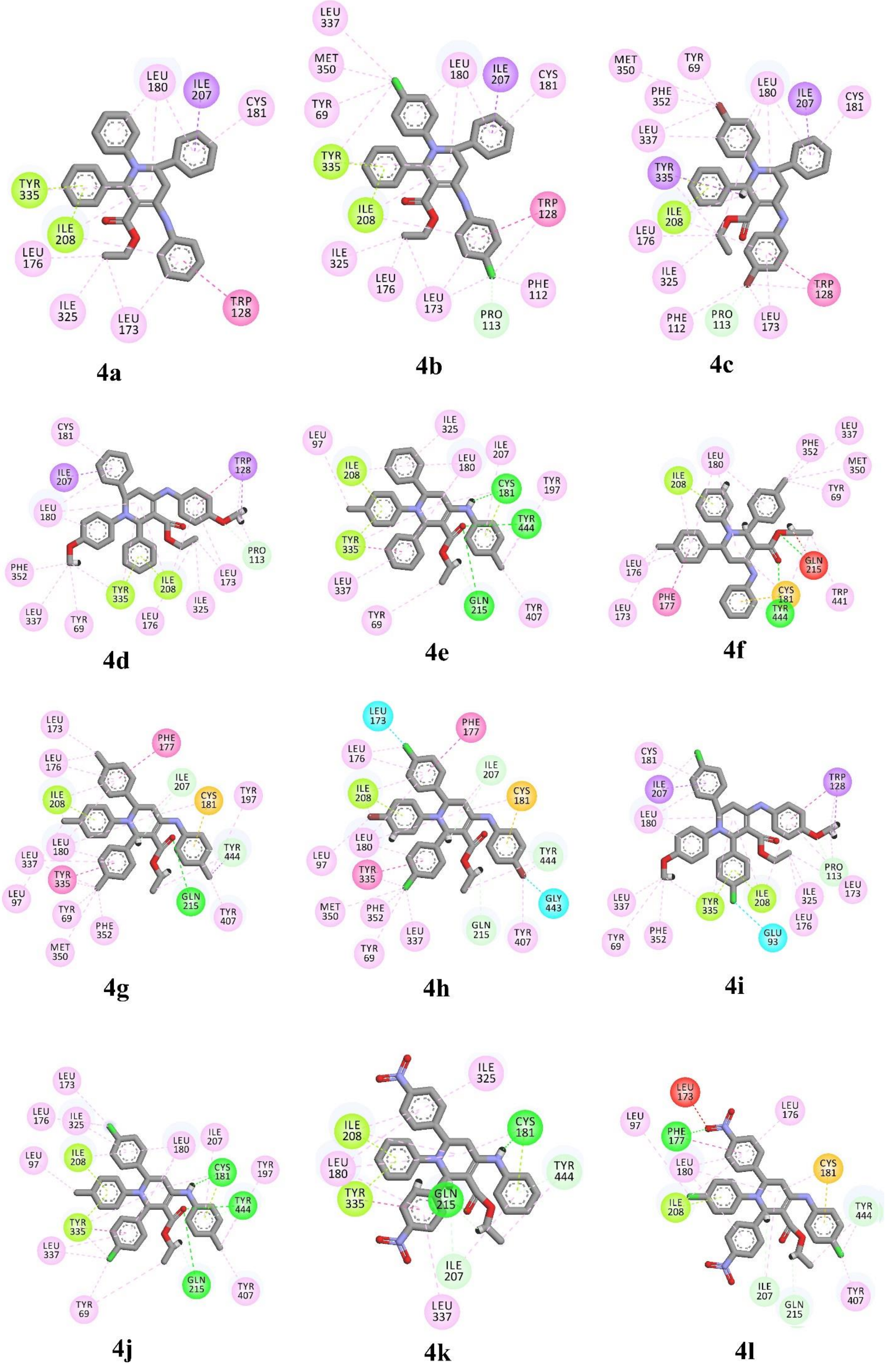

Figure S1. 2D representation of the predicted binding mode for the synthesized tetrahydropyridines (**4a**–**4o**) against MAO-A enzyme.

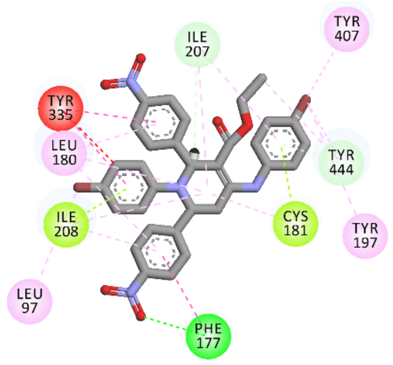

4m

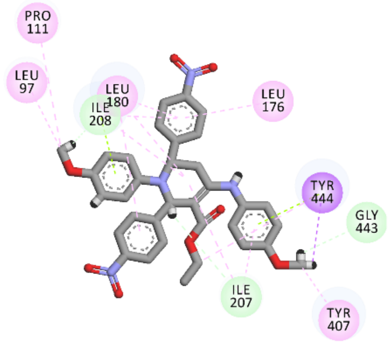

4n

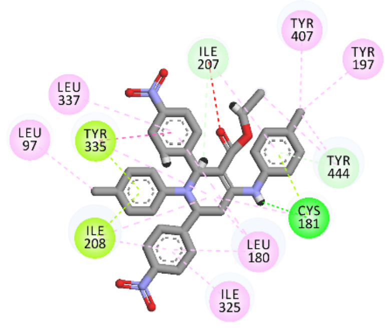

4o

Interactions

- Conventional Hydrogen Bond
- Carbon Hydrogen Bond
- Pi-Donor Hydrogen Bond
- Pi-Lone Pair
- Pi-Pi T-shaped
- Pi-Pi Stacked
- Pi-Alkyl
- Alkyl
- Unfavorable Acceptor-Acceptor
- Halogen (Cl, Br, I)
- Pi-Sigma
- Pi-Sulfur

Figure S1. *Continued.*

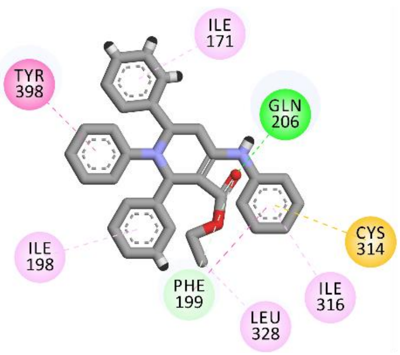

4a

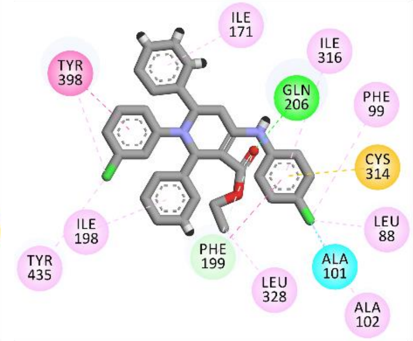

4b

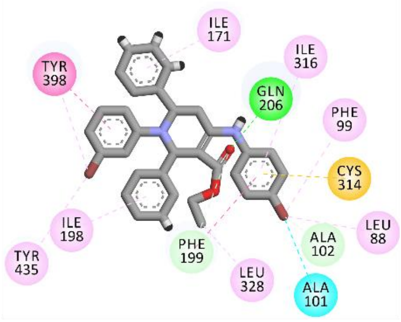

4c

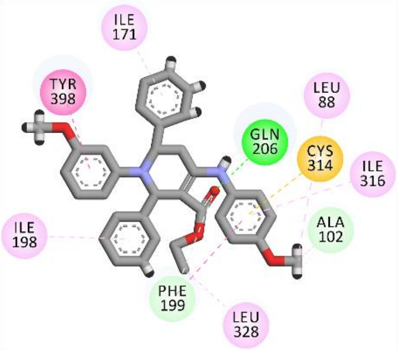

4d

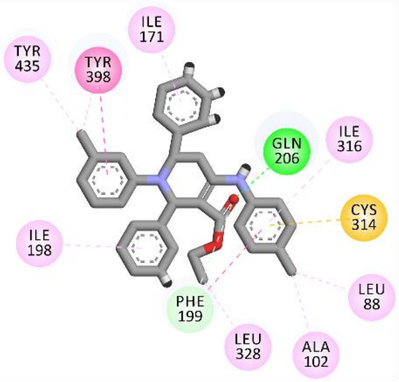

4e

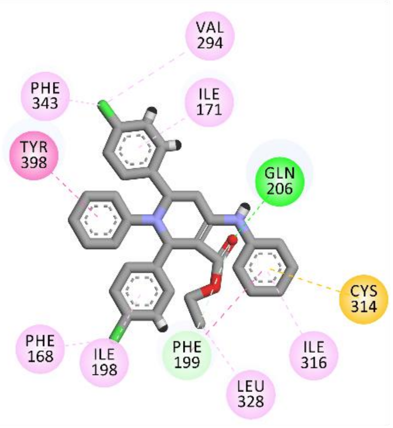

4f

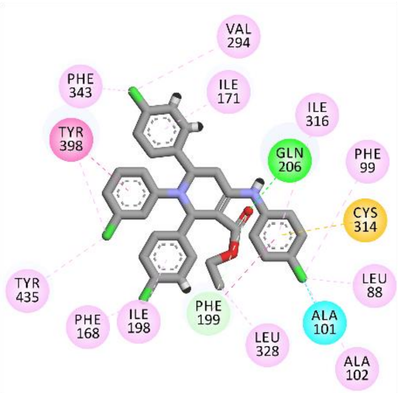

4g

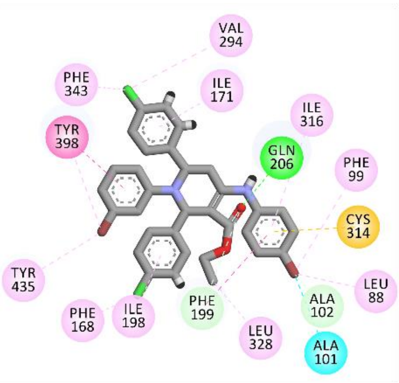

4h

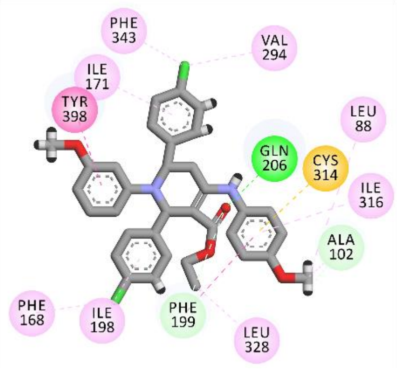

4i

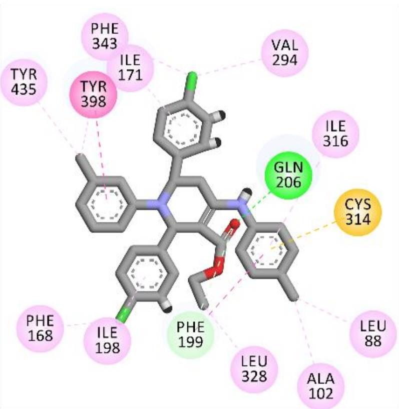

4j

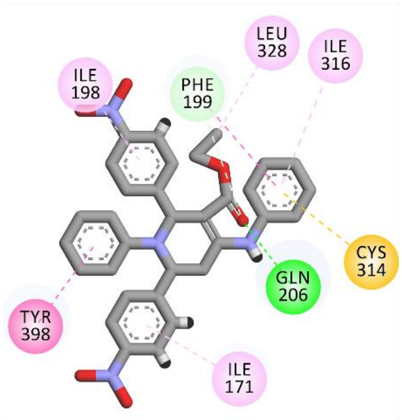

4k

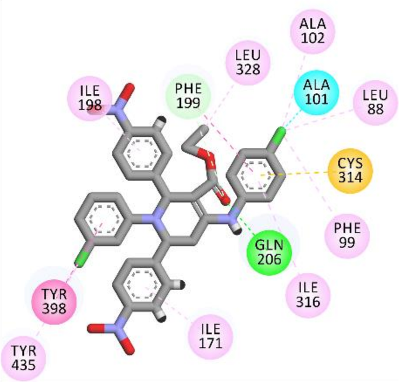

4l

Figure S2. 2D representation of the predicted binding mode for the synthesized tetrahydropyridines (**4a**–**4o**) against MAO-B enzyme.

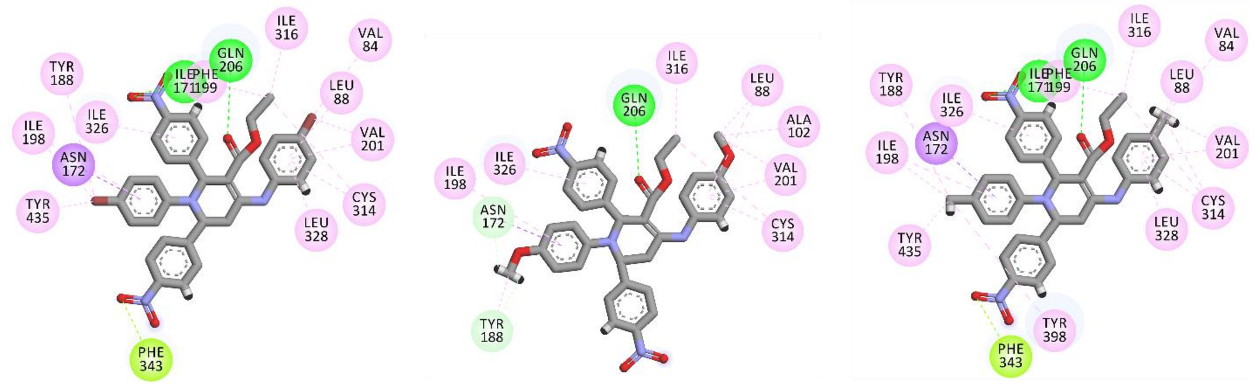

4m **4n** **4o**

Interactions

- Conventional Hydrogen Bond
- Carbon Hydrogen Bond
- Pi-Lone Pair
- Pi-Sulfur
- Pi-Pi T-shaped
- Pi-Pi Stacked
- Pi-Alkyl
- Pi-Sigma
- Alkyl
- Halogen (Cl, Br, I)

Figure S2. *Continued.*

Figure S3. ^{1}H- and ^{13}C-NMR spectra of compounds 4a-4o

^{1}H-NMR of 4a

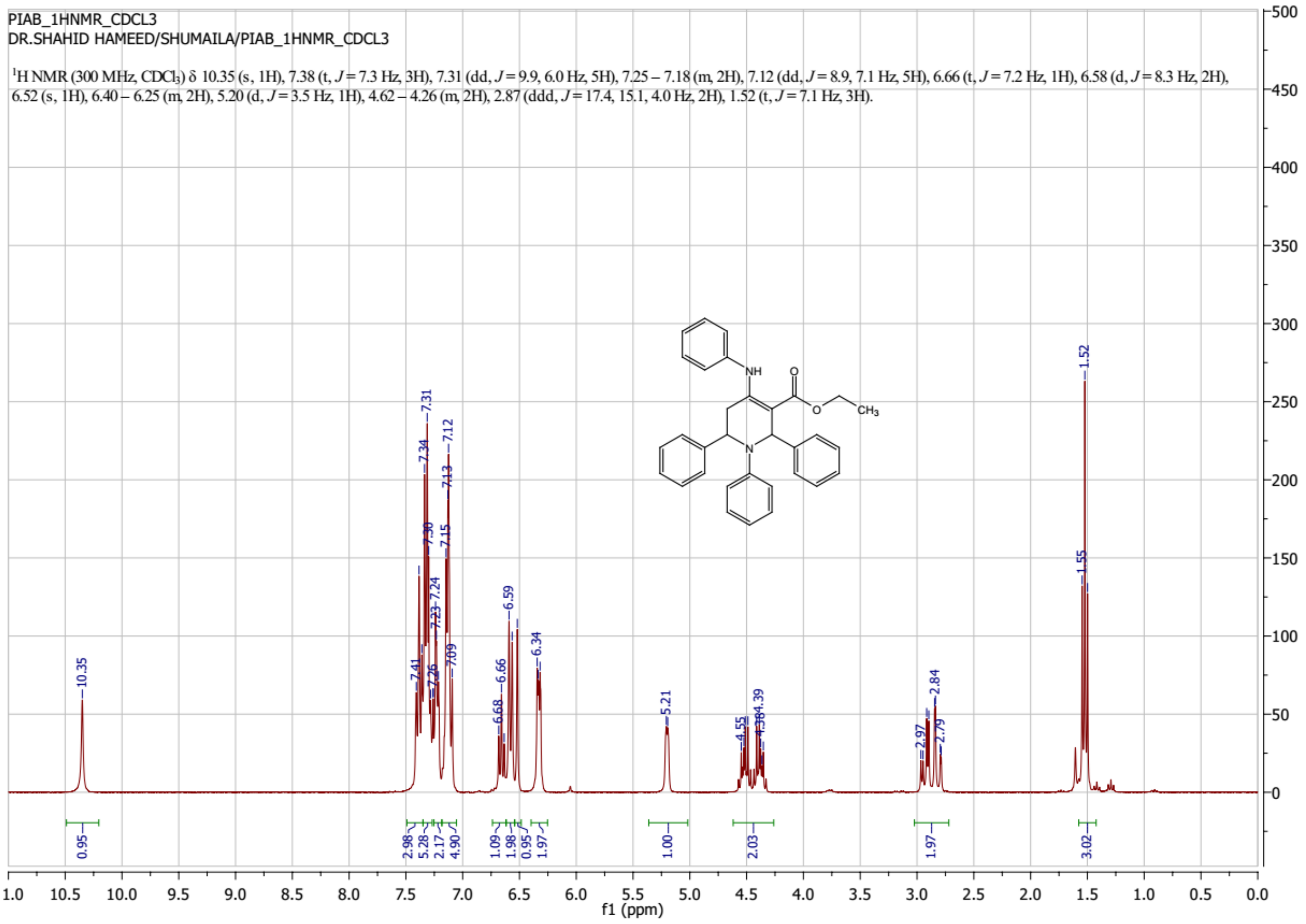

^{13}C-NMR of 4a

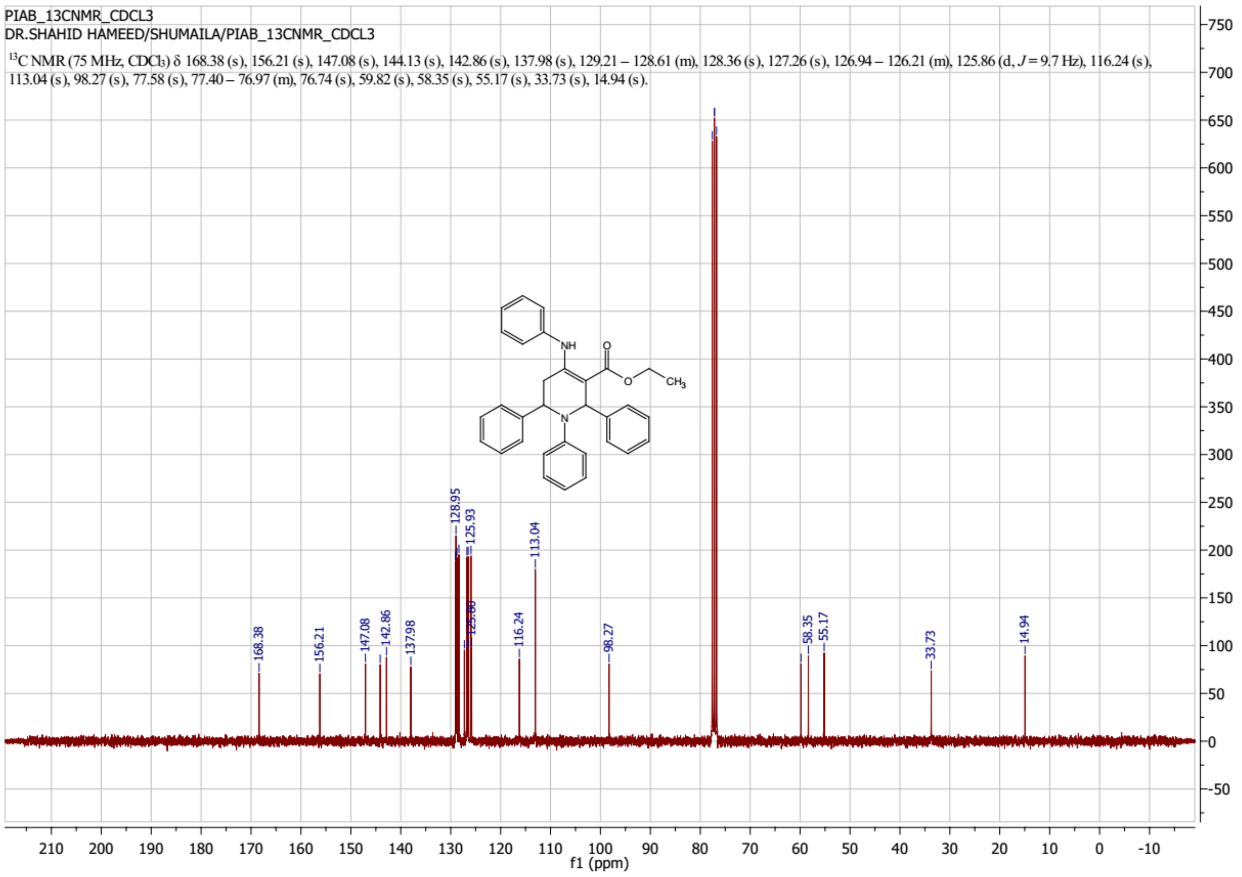

^{1}H-NMR of 4b

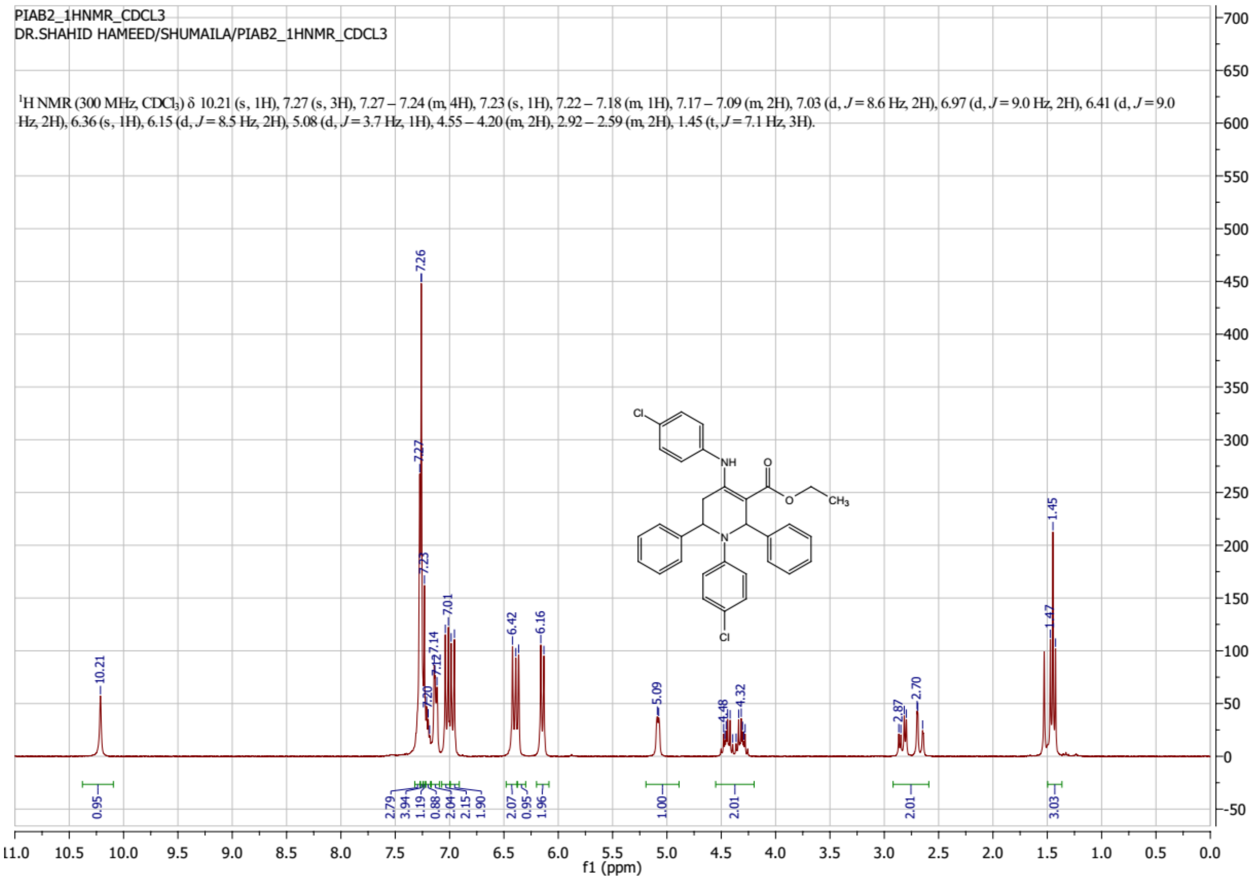

^{13}C-NMR of 4b

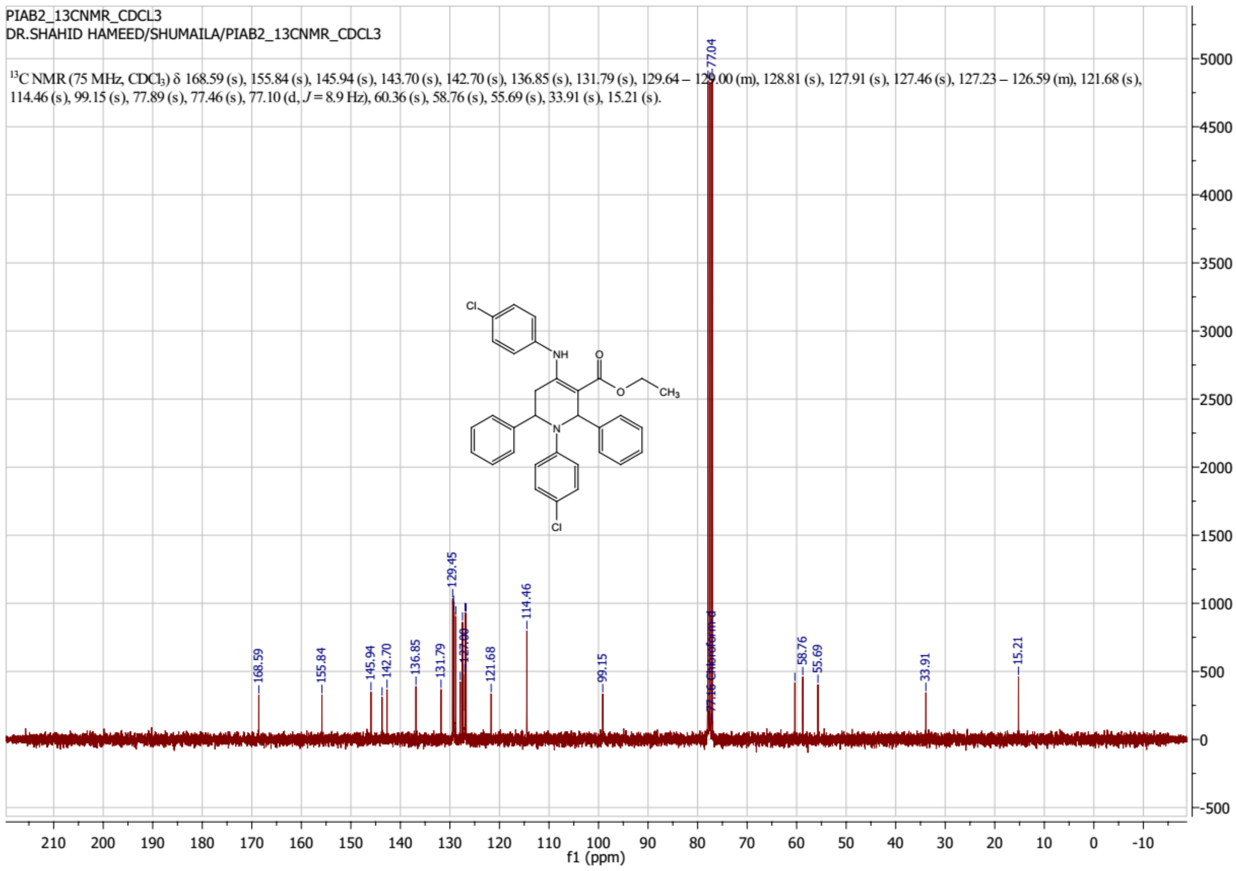

^{1}H-NMR of 4c

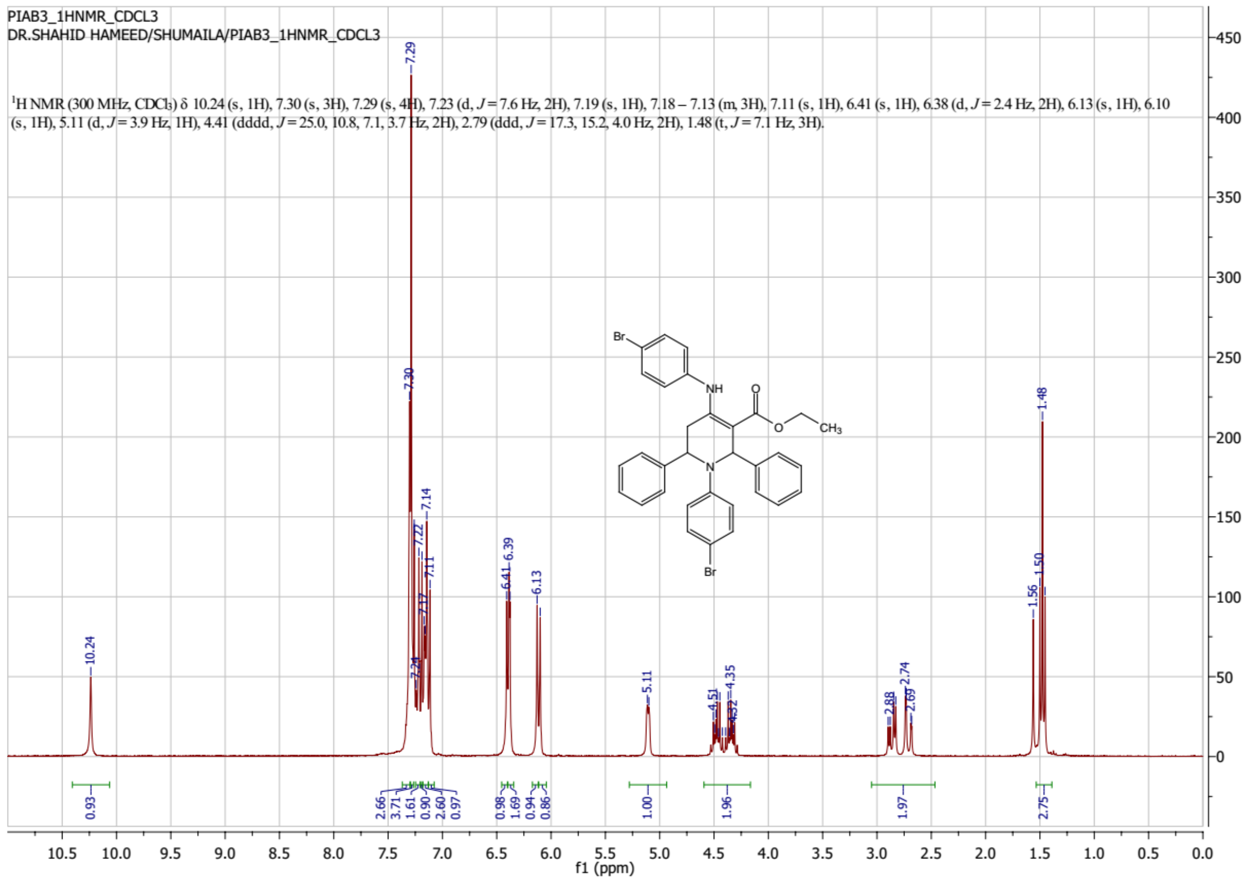

^{13}C-NMR of 4c

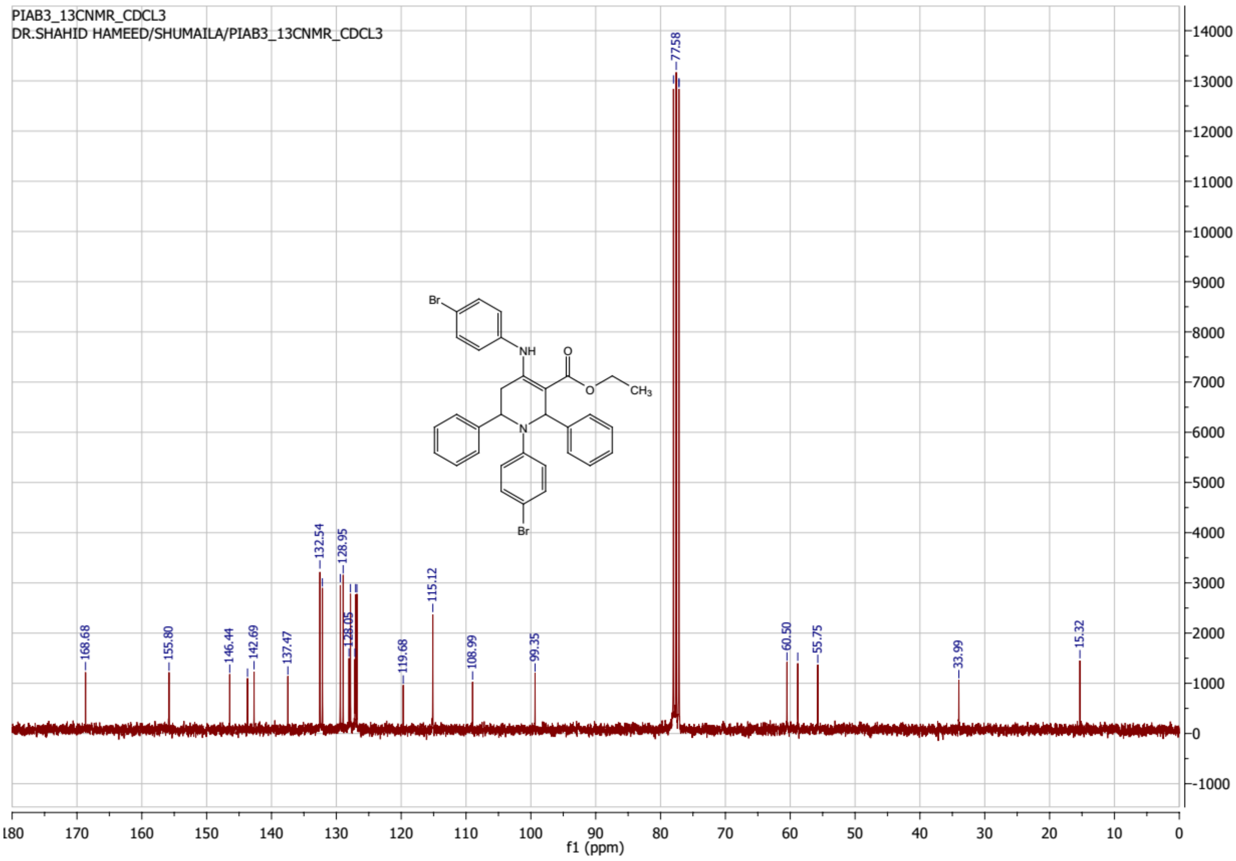

^{1}H-NMR of 4d

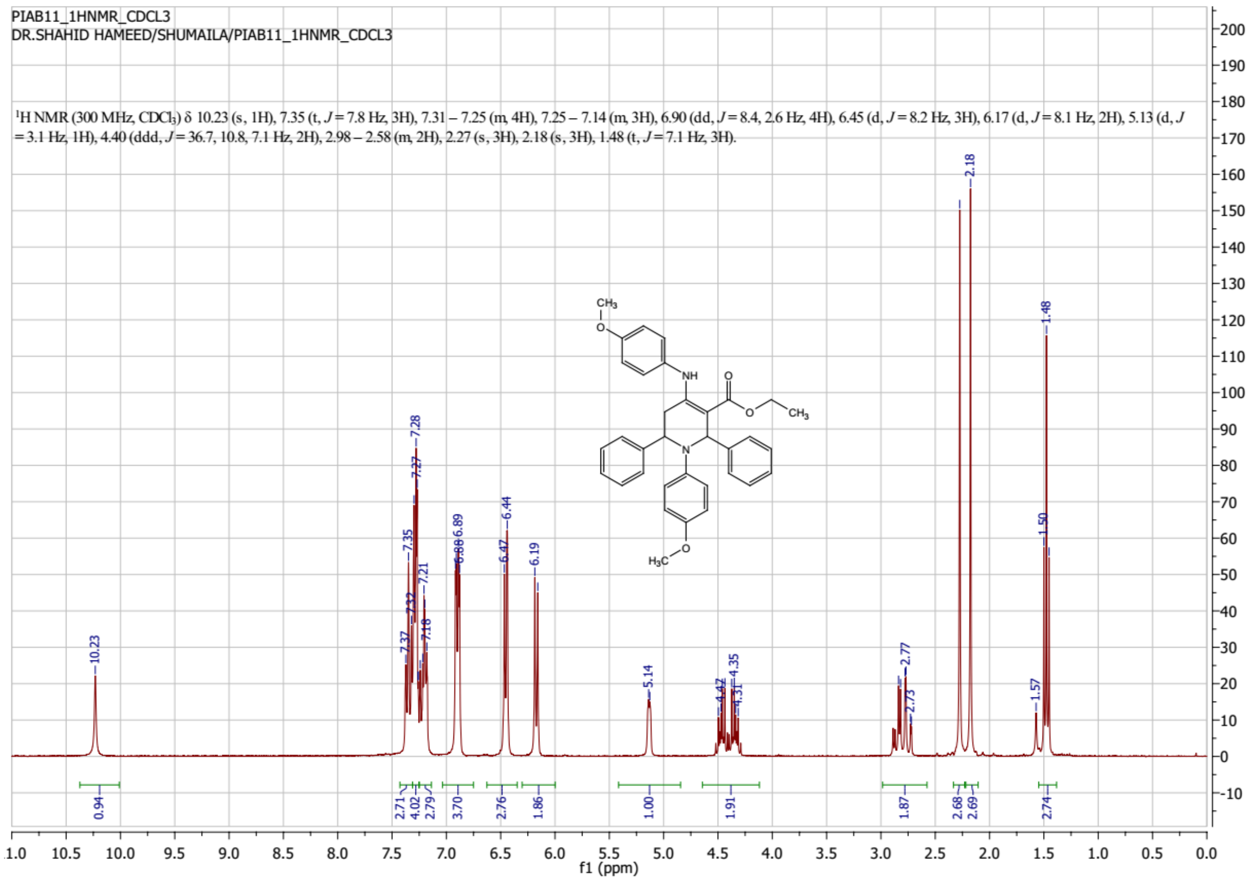

^{13}C-NMR of 4d

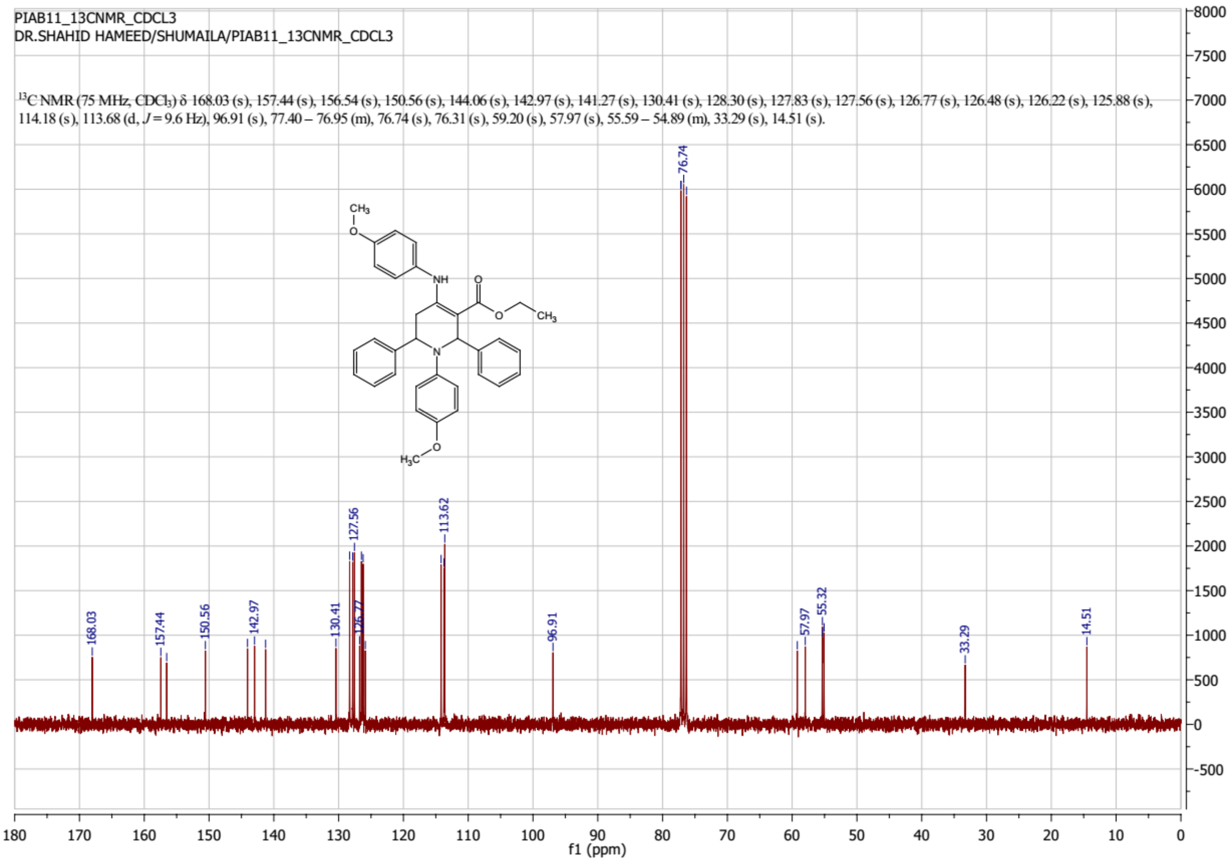

^{1}H-NMR of 4e

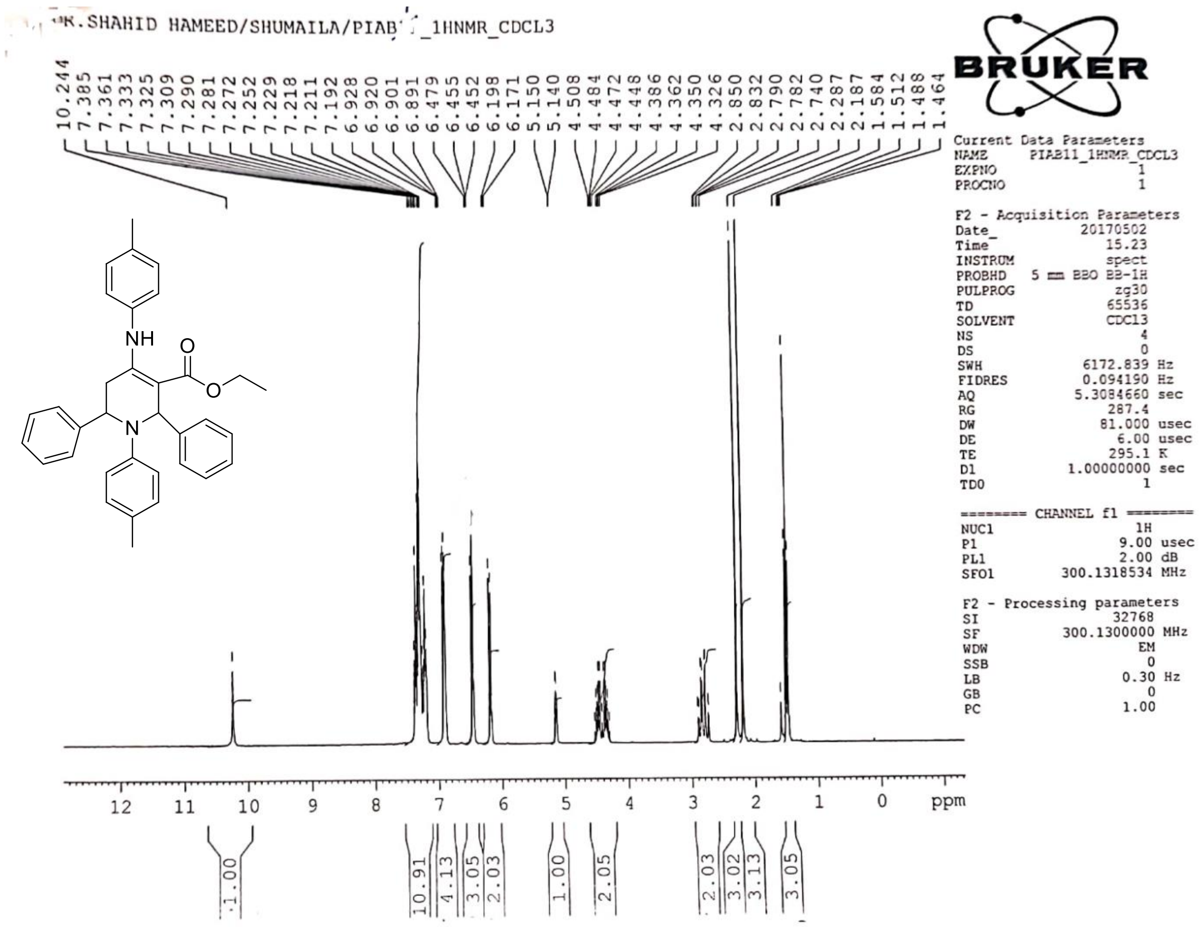

^{13}C-NMR Spectra of 4e

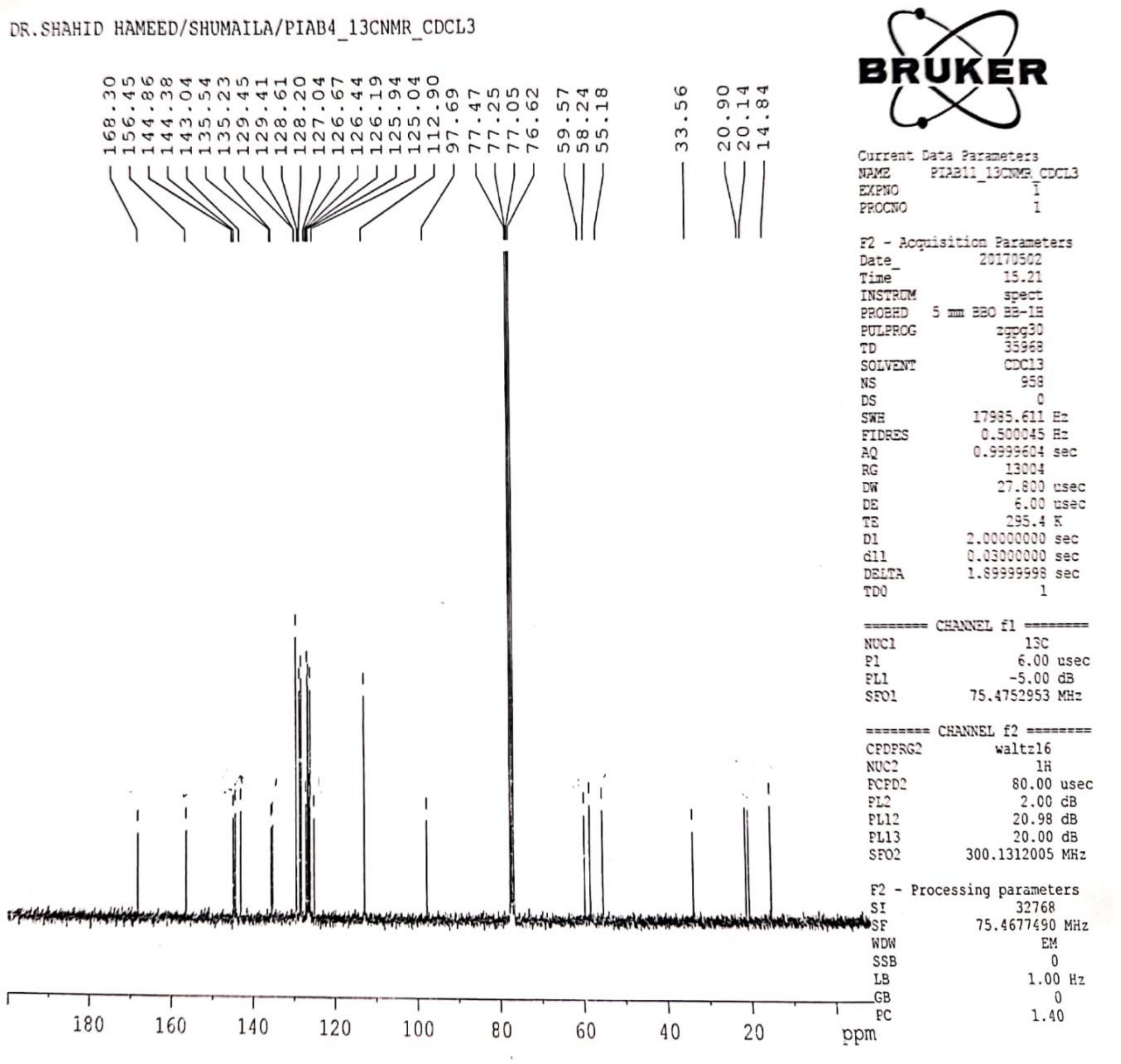

^{1}H-NMR of 4f

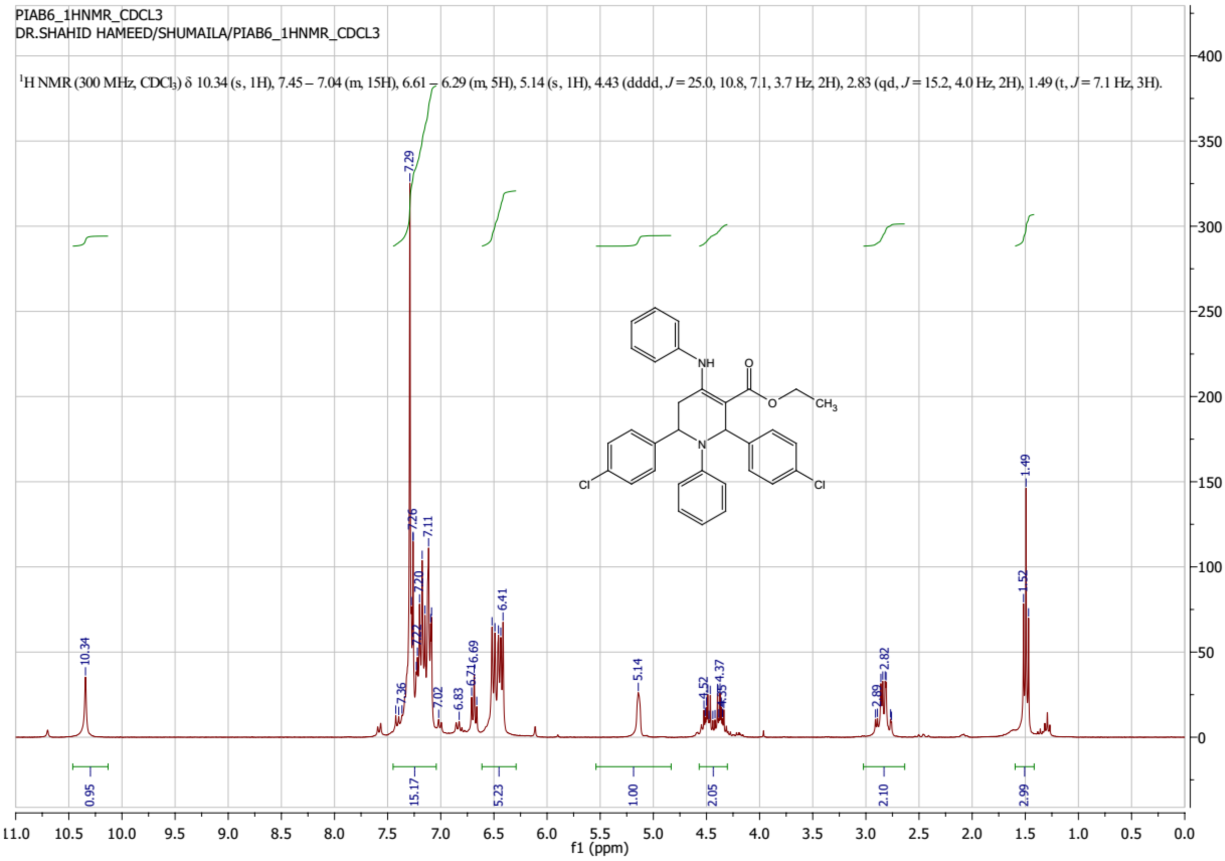

^{13}C-NMR of 4f

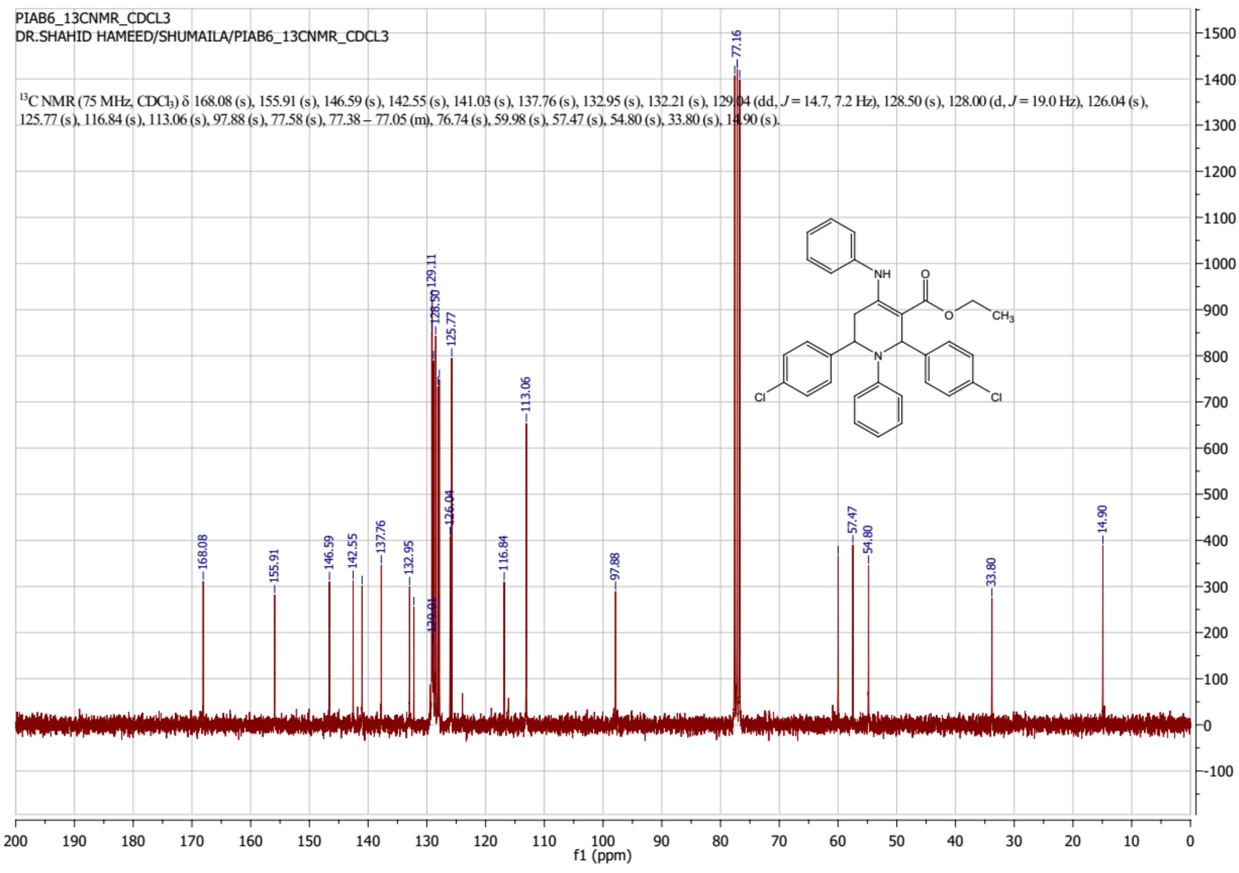

^{1}H-NMR of 4g

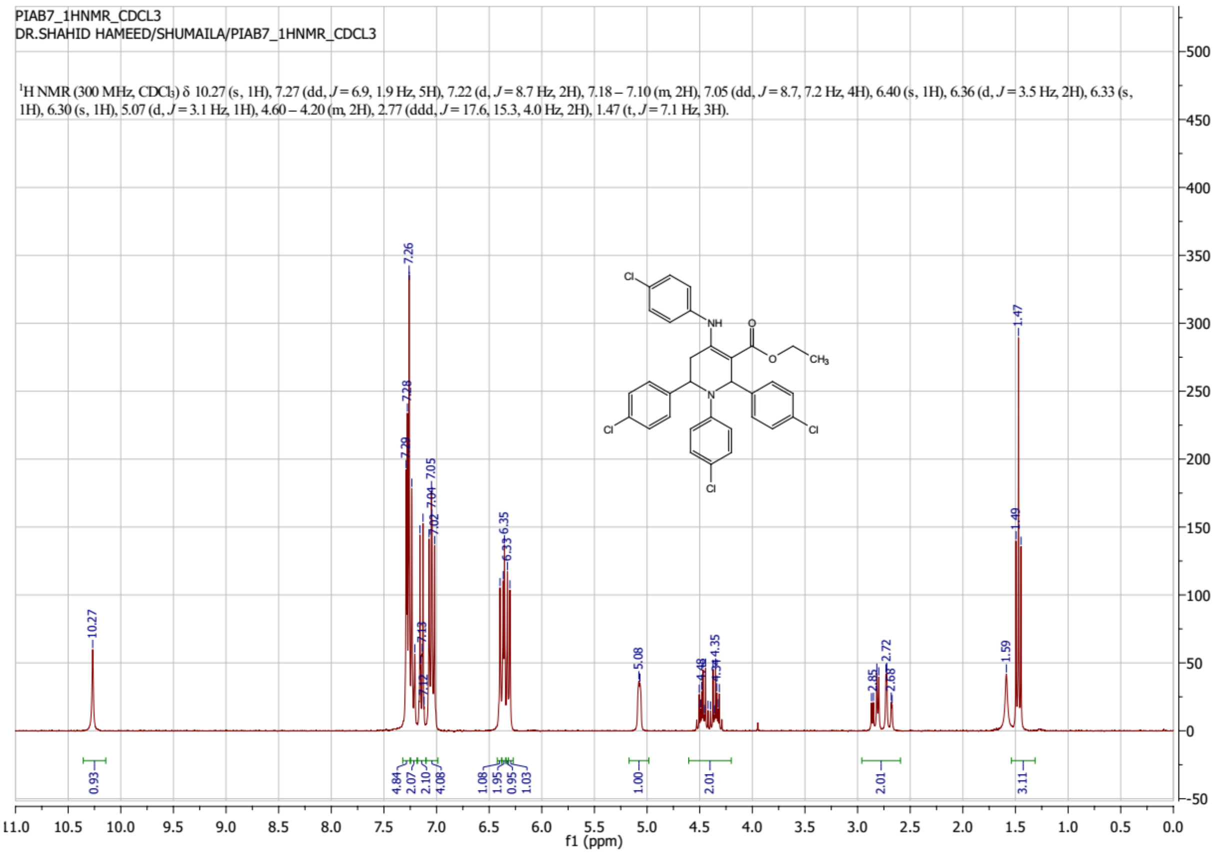

^{13}C-NMR of 4g

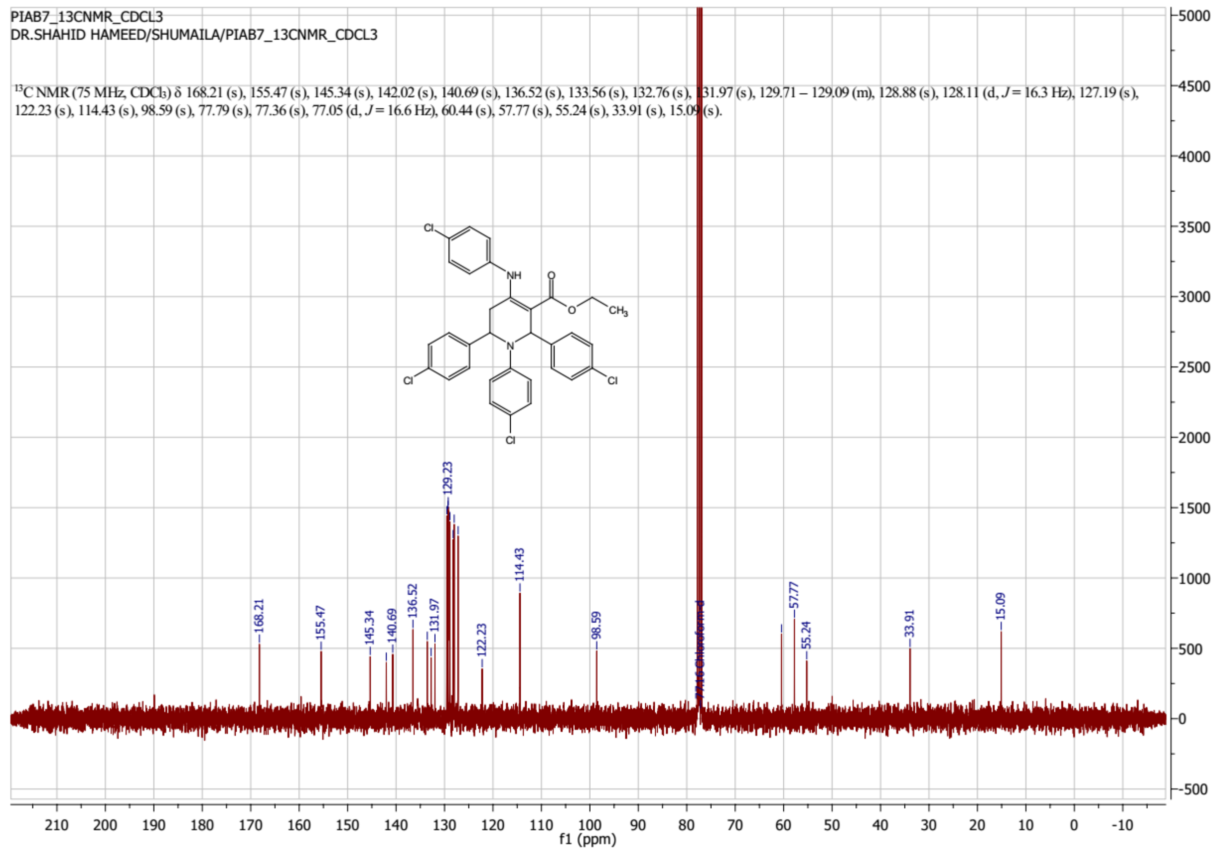

^{1}H-NMR of 4h

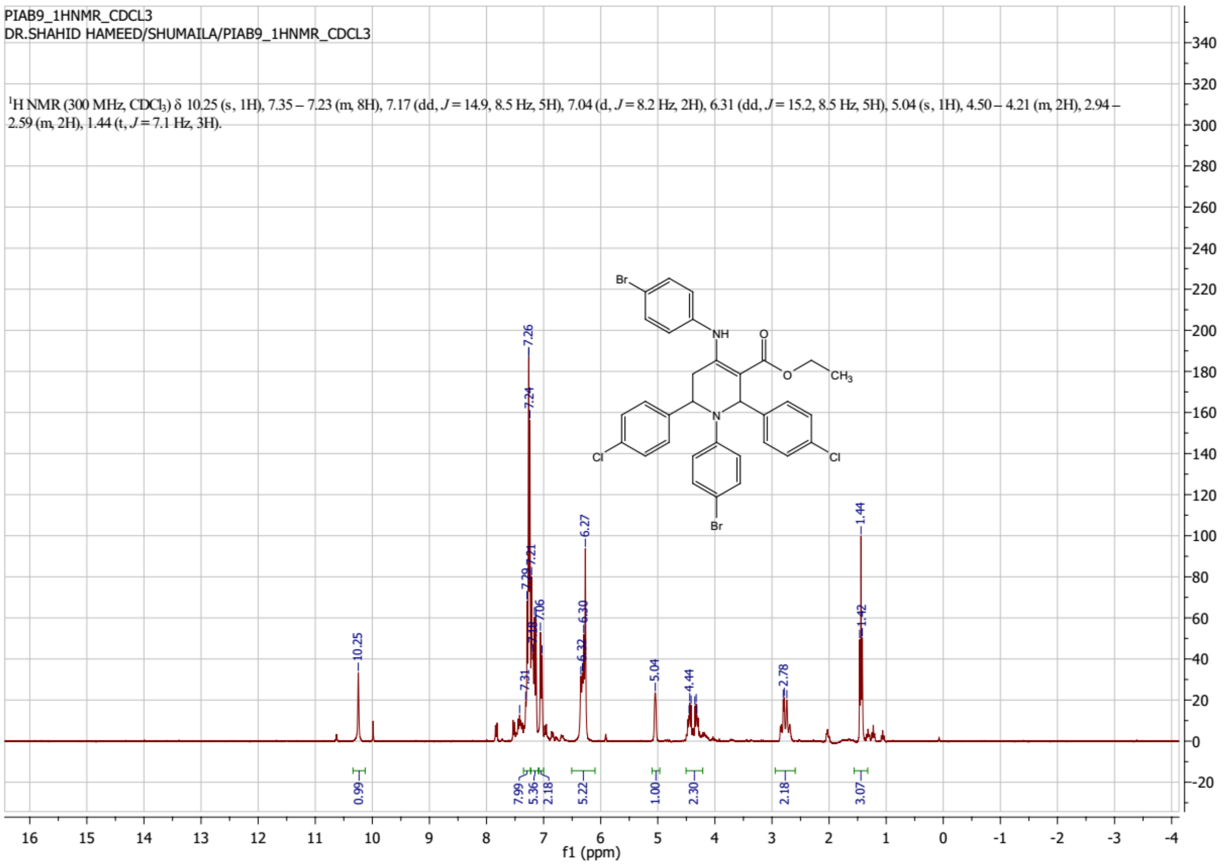

^{13}C-NMR of 4h

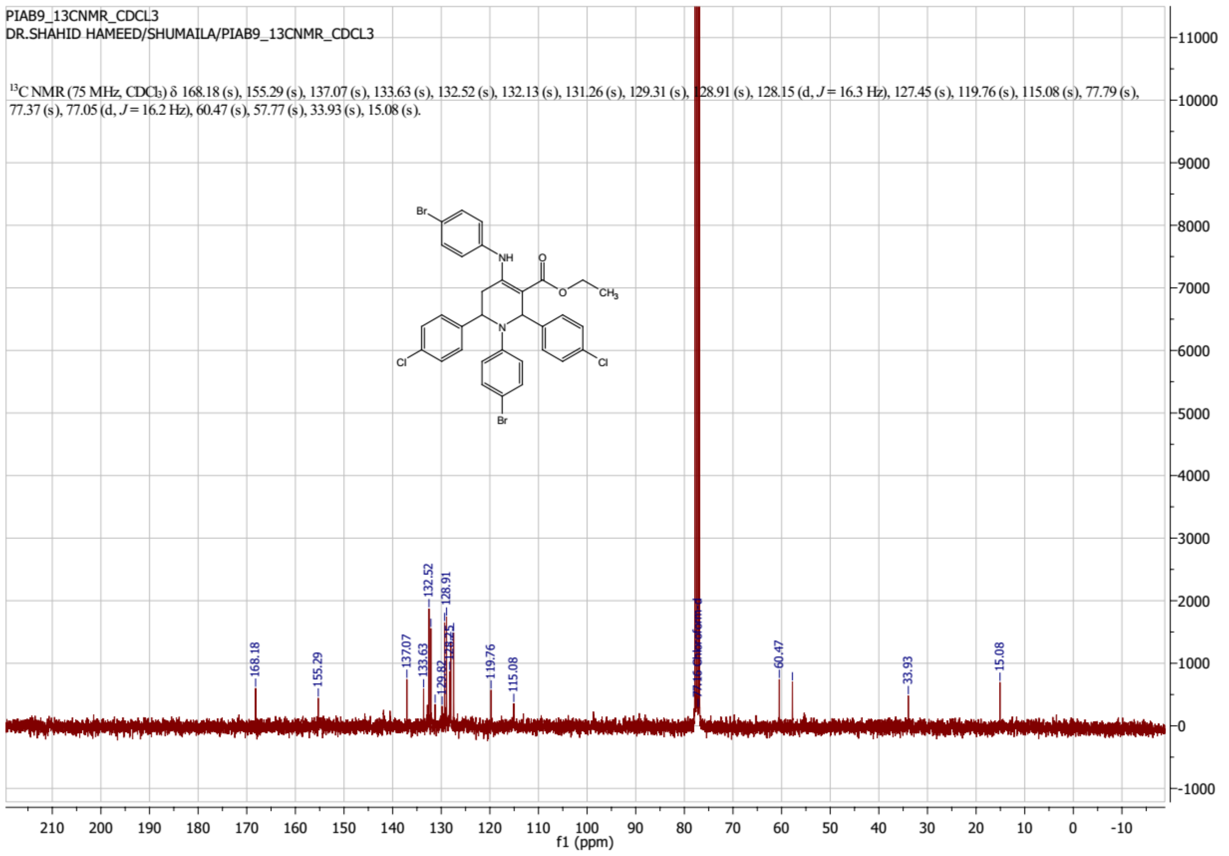

^{1}H NMR of 4i

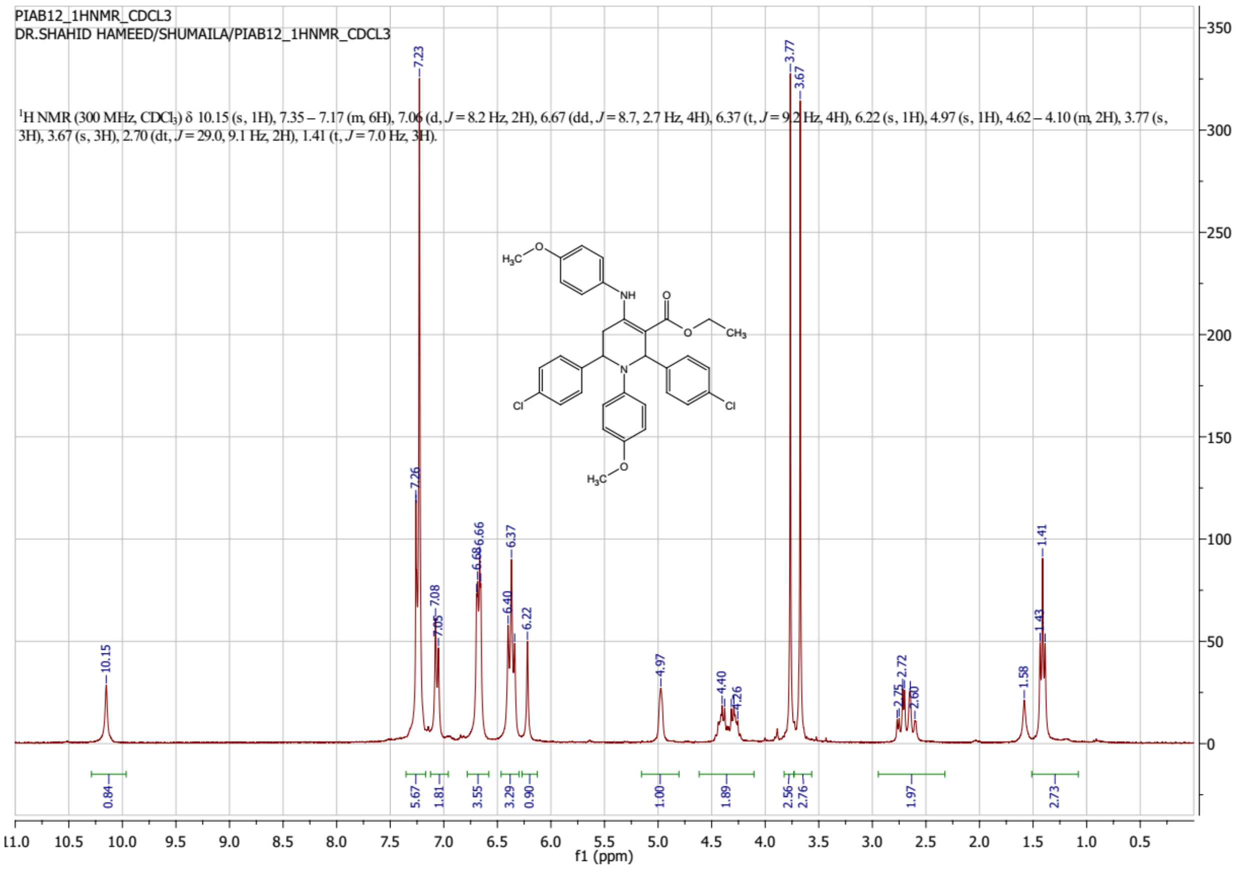

^{13}C-NMR of 4i

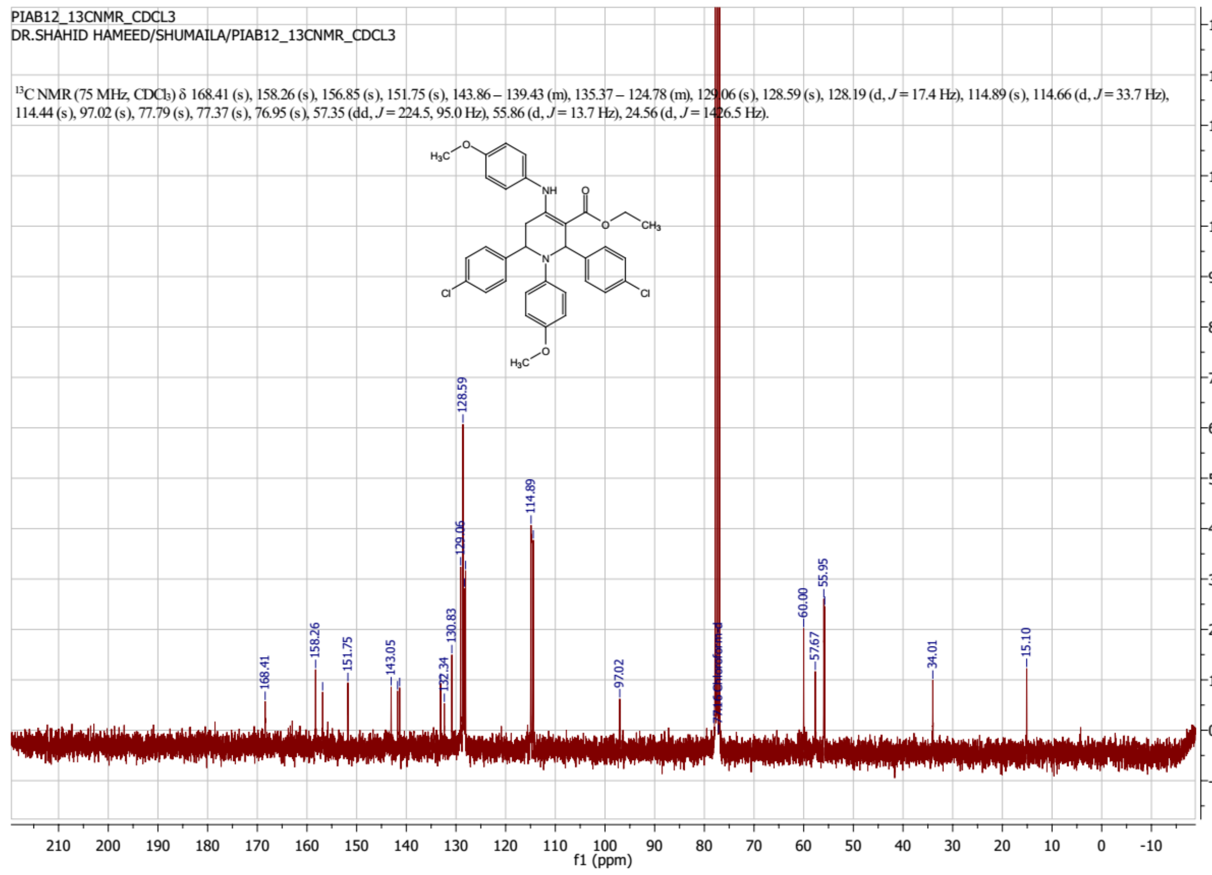

^{1}H NMR of 4j

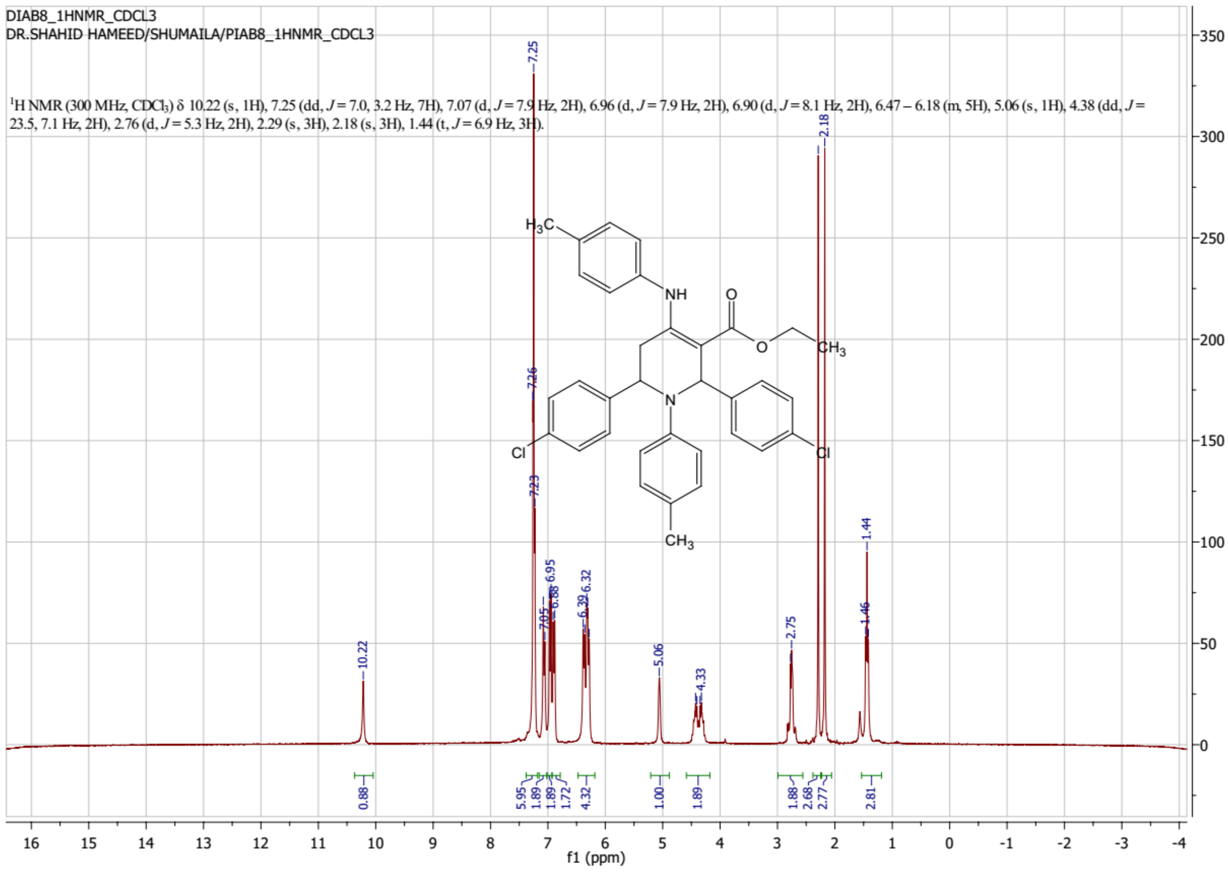

^{13}C-NMR of 4j

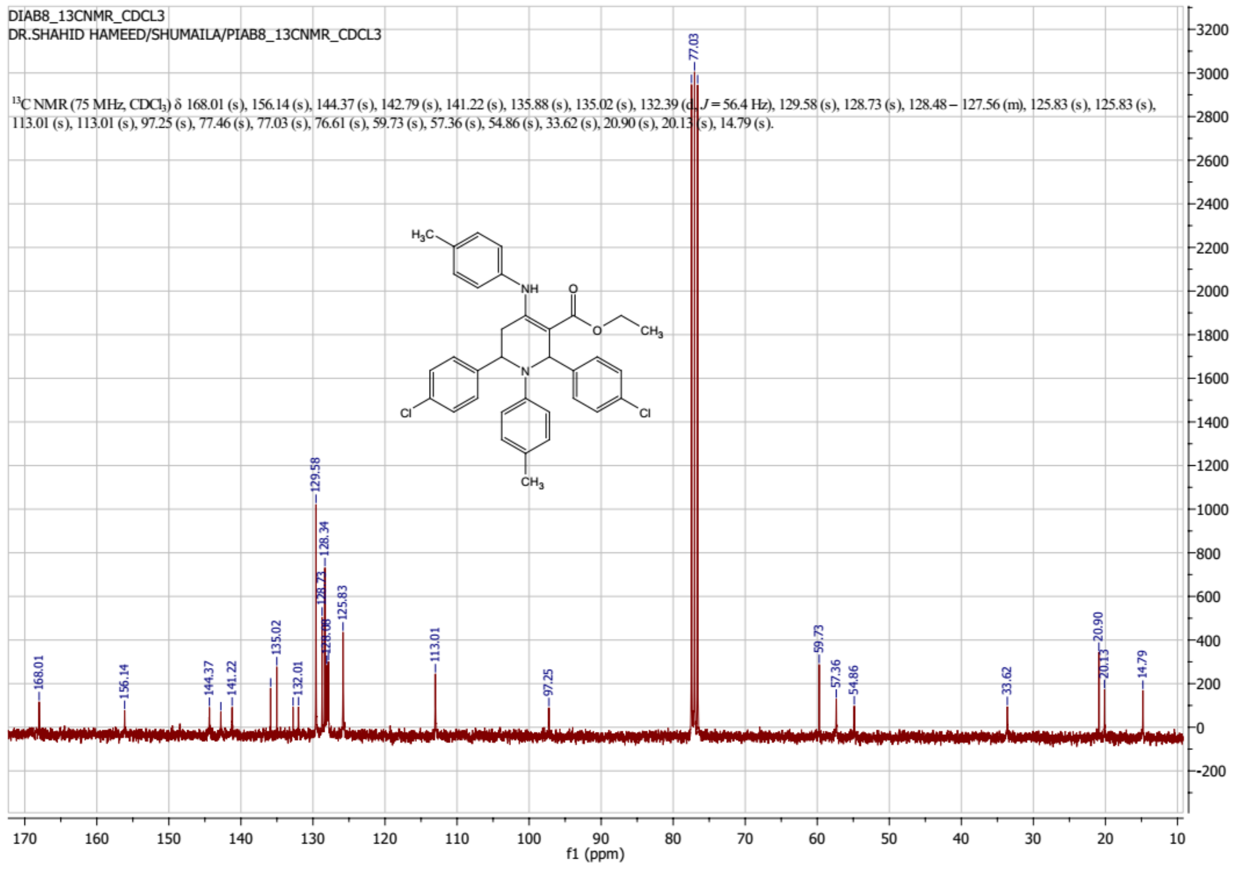

^{1}H NMR of 4k

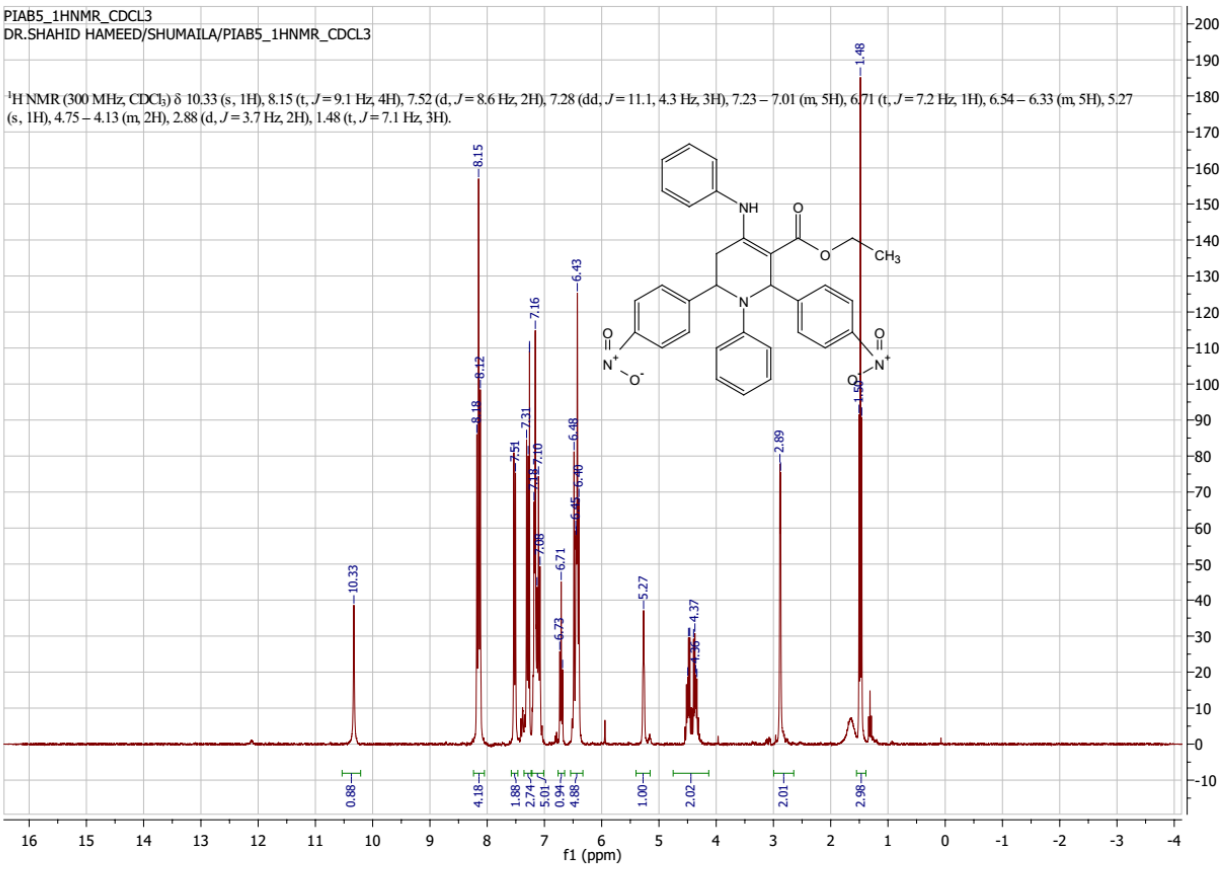

^{13}C-NMR of 4k

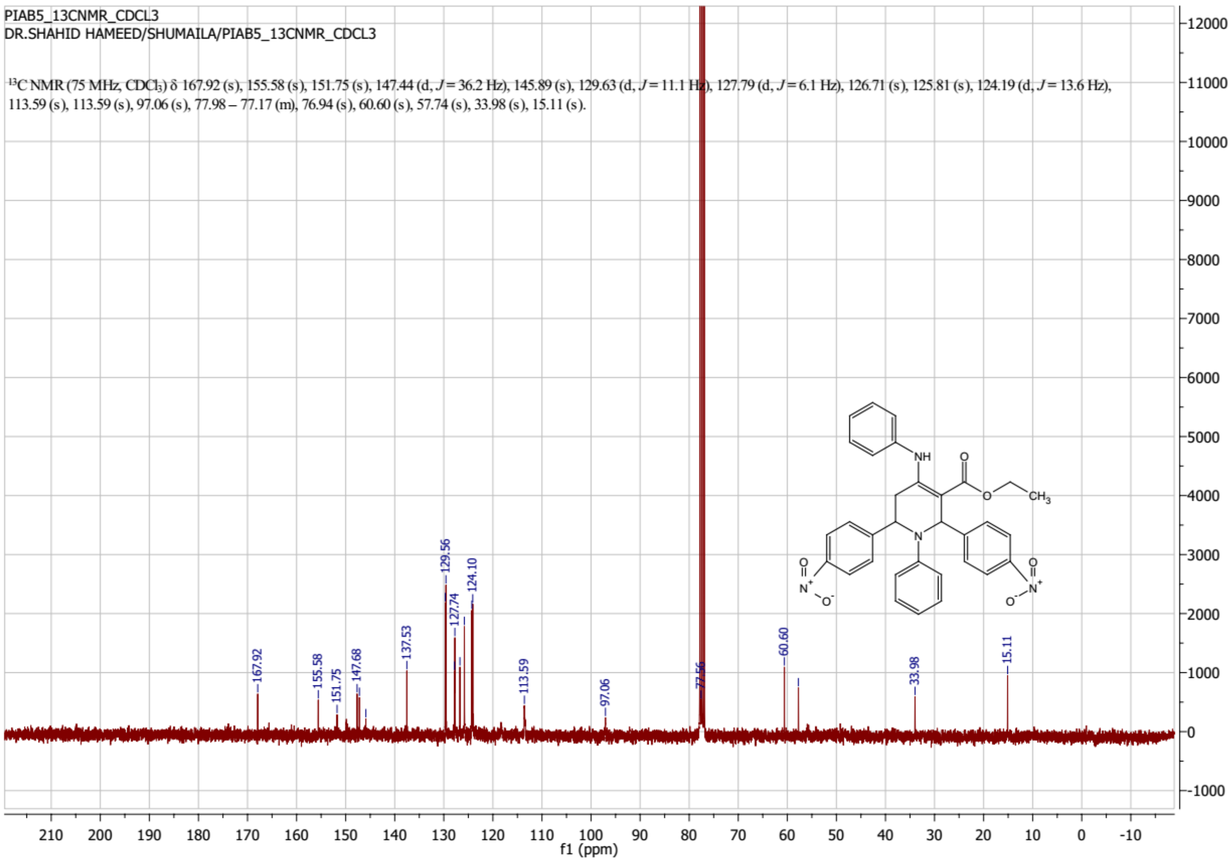

^{1}H NMR of 4l

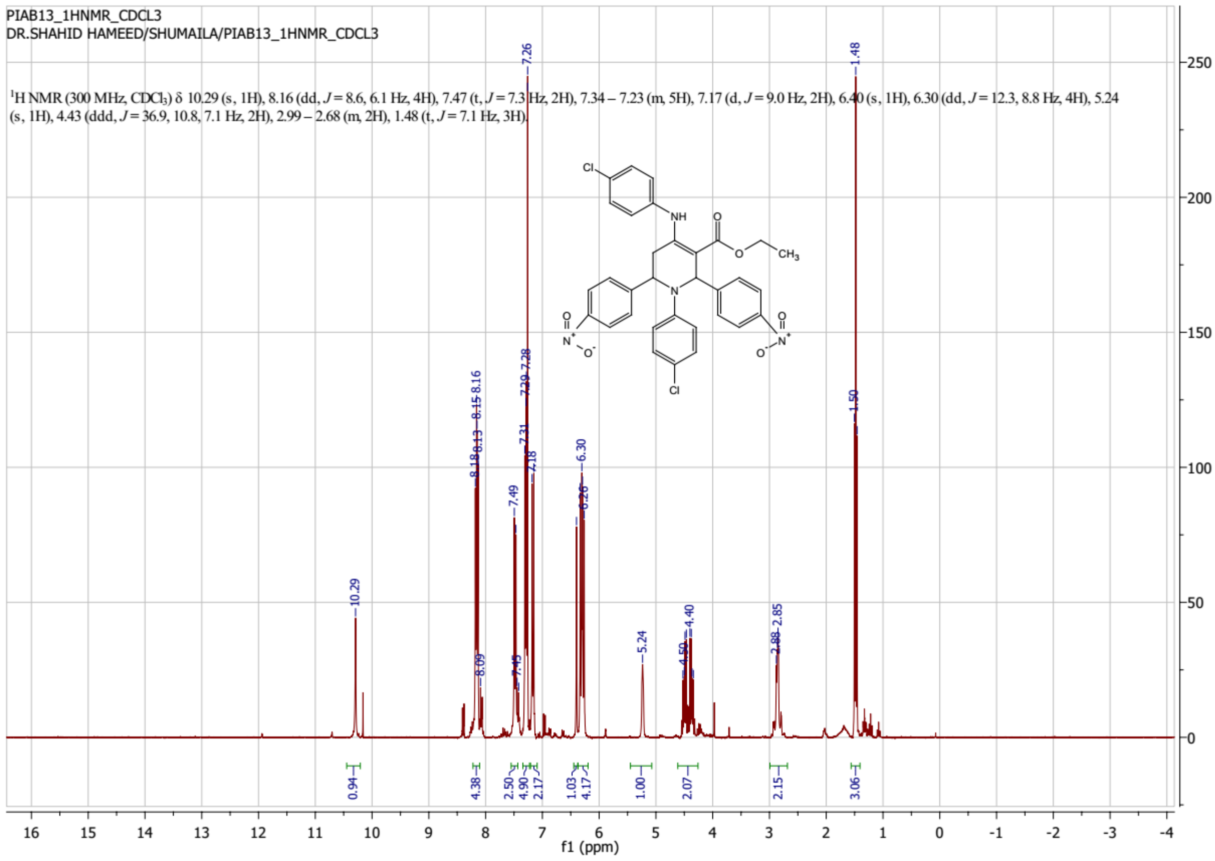

^{13}C-NMR of 4l

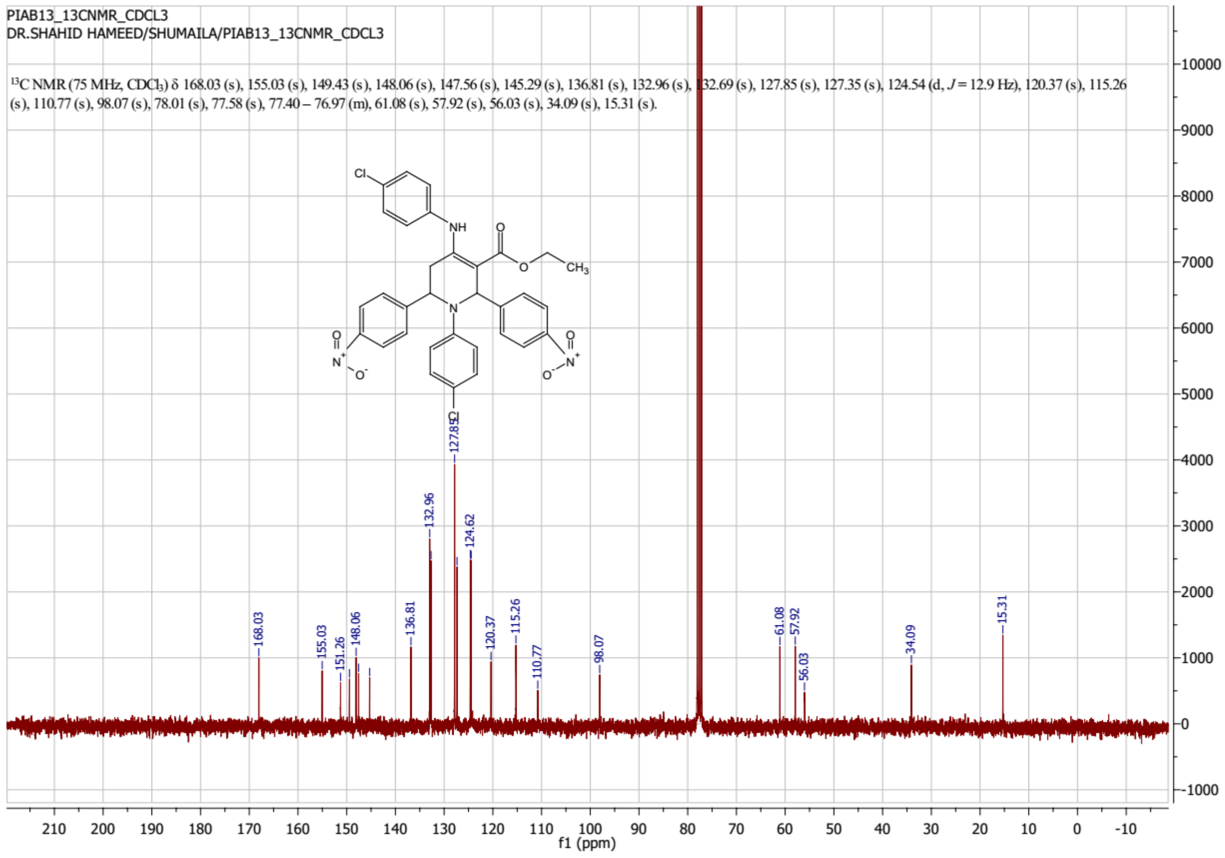

^{1}H NMR of 4m

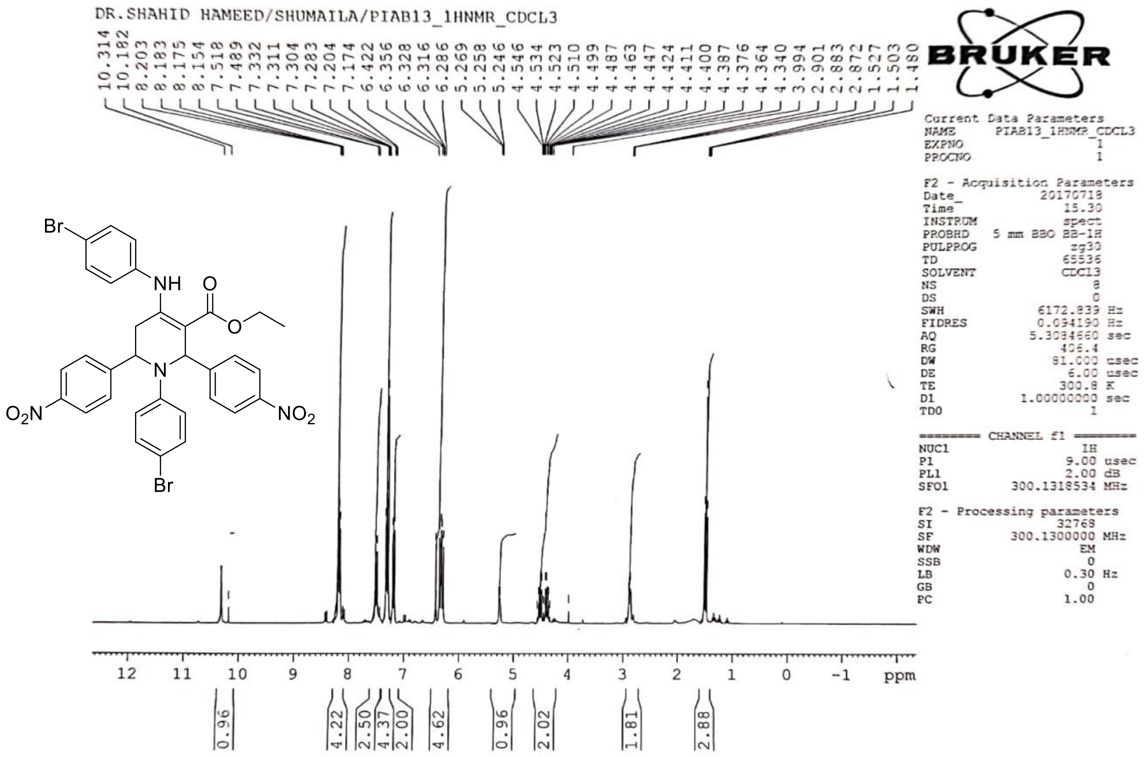

^{13}C-NMR of 4m

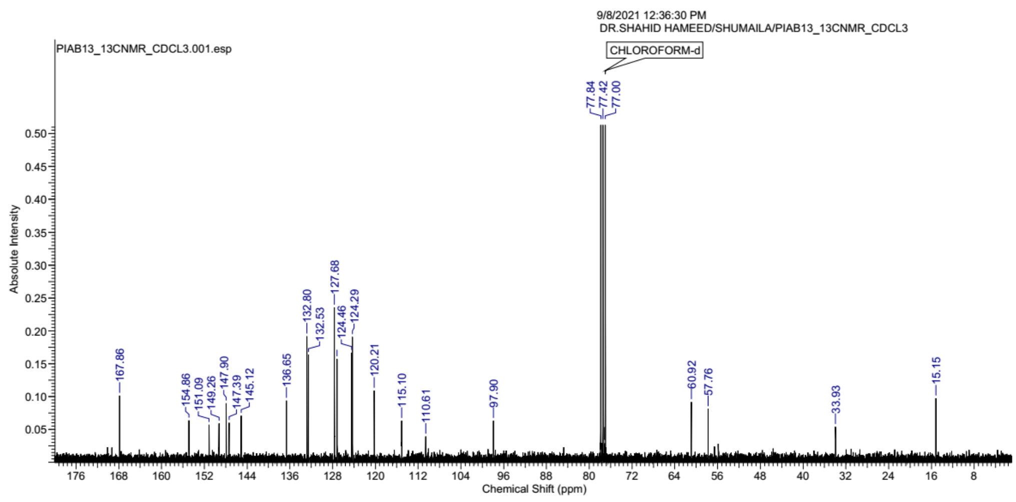

^{1}H NMR of 4n

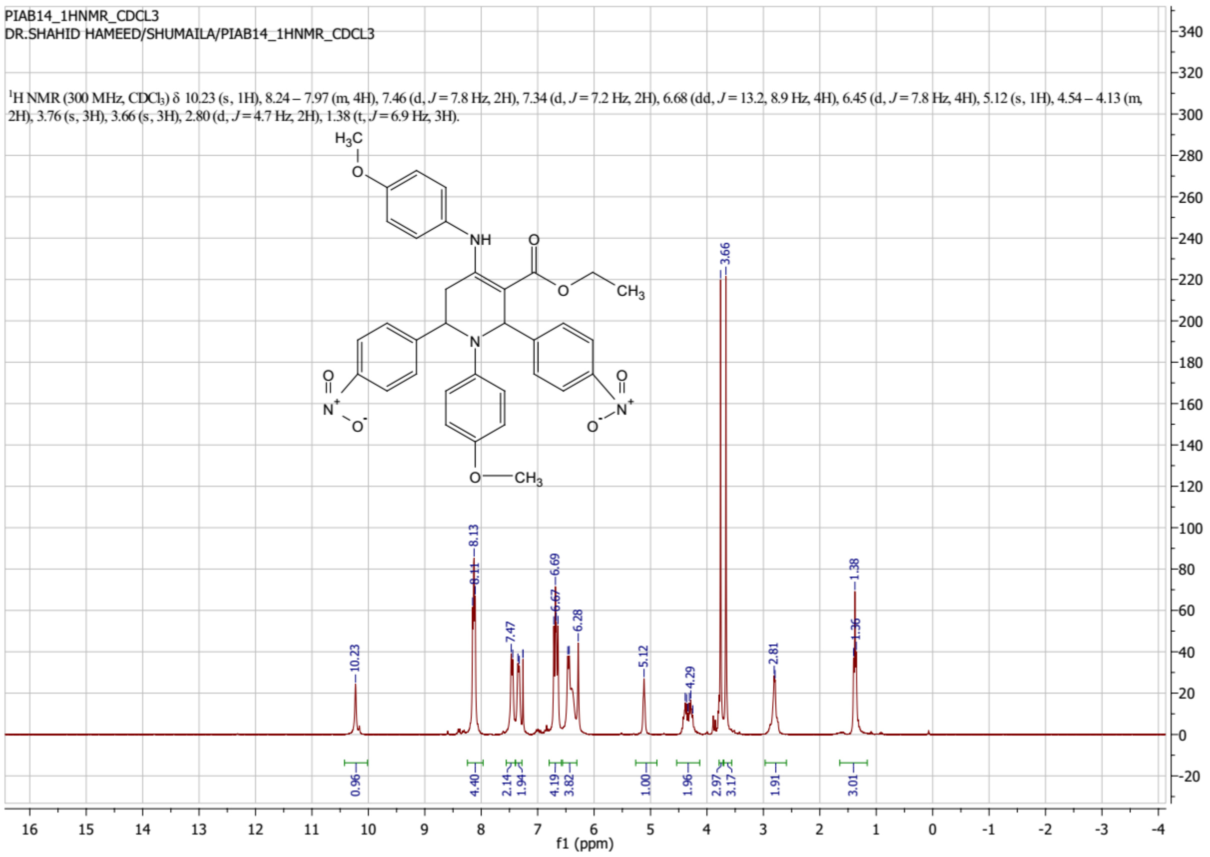

^{13}C-NMR of 4n

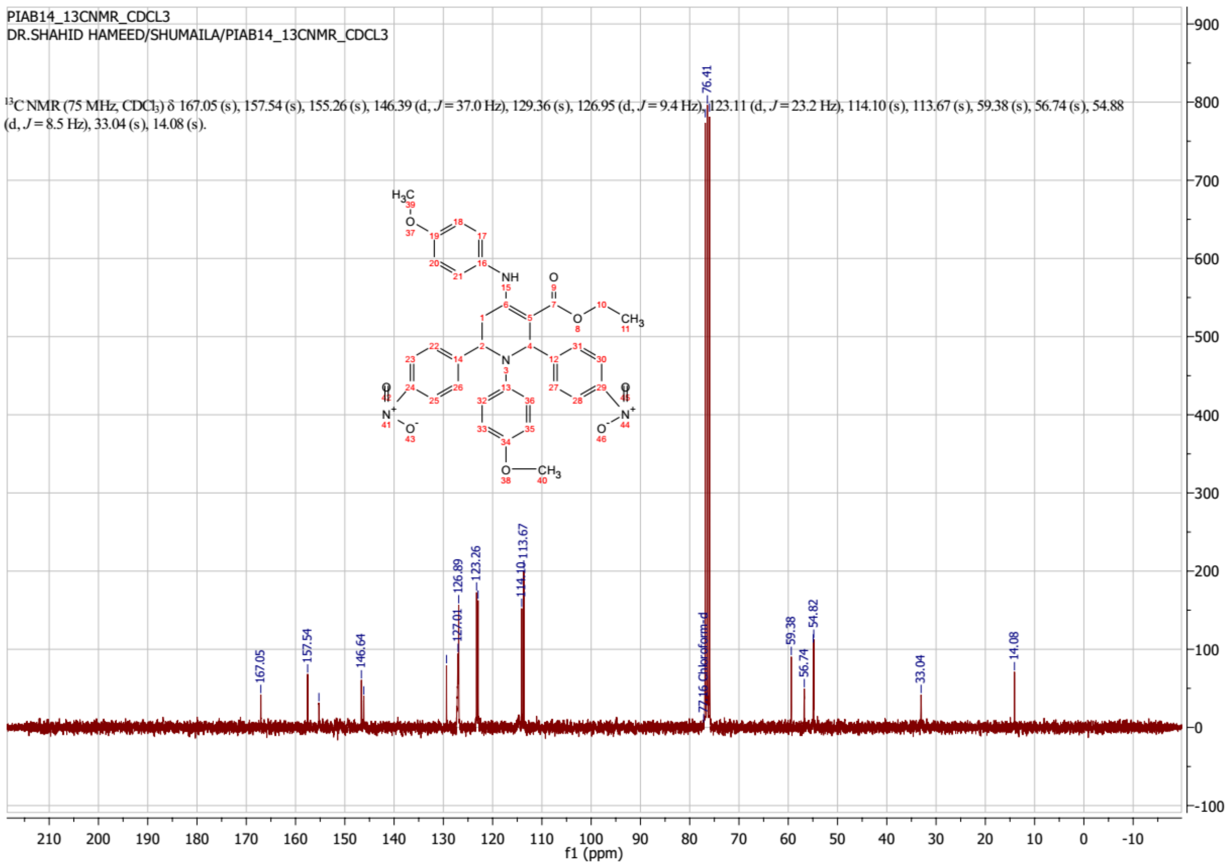

^{1}H NMR of 4o

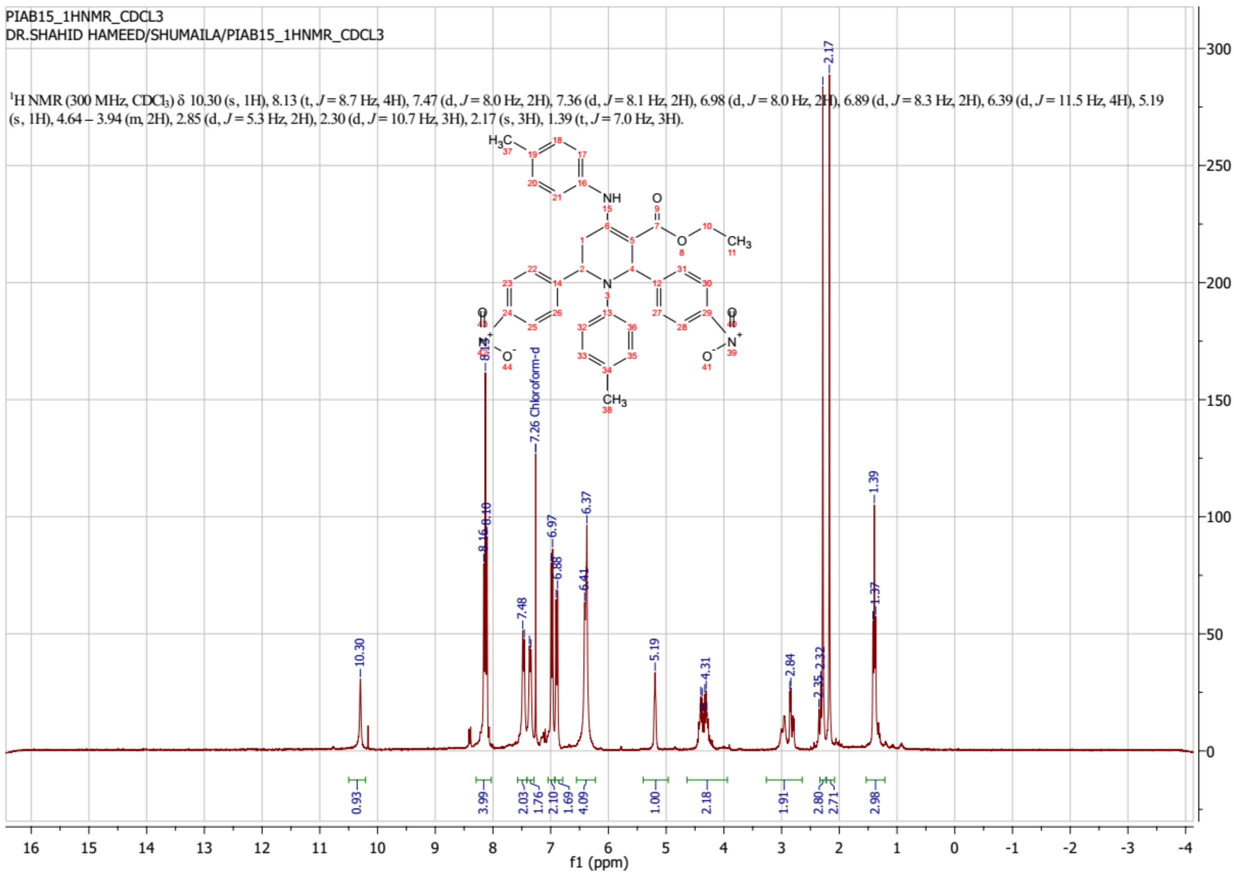

^{13}C-NMR of 4o

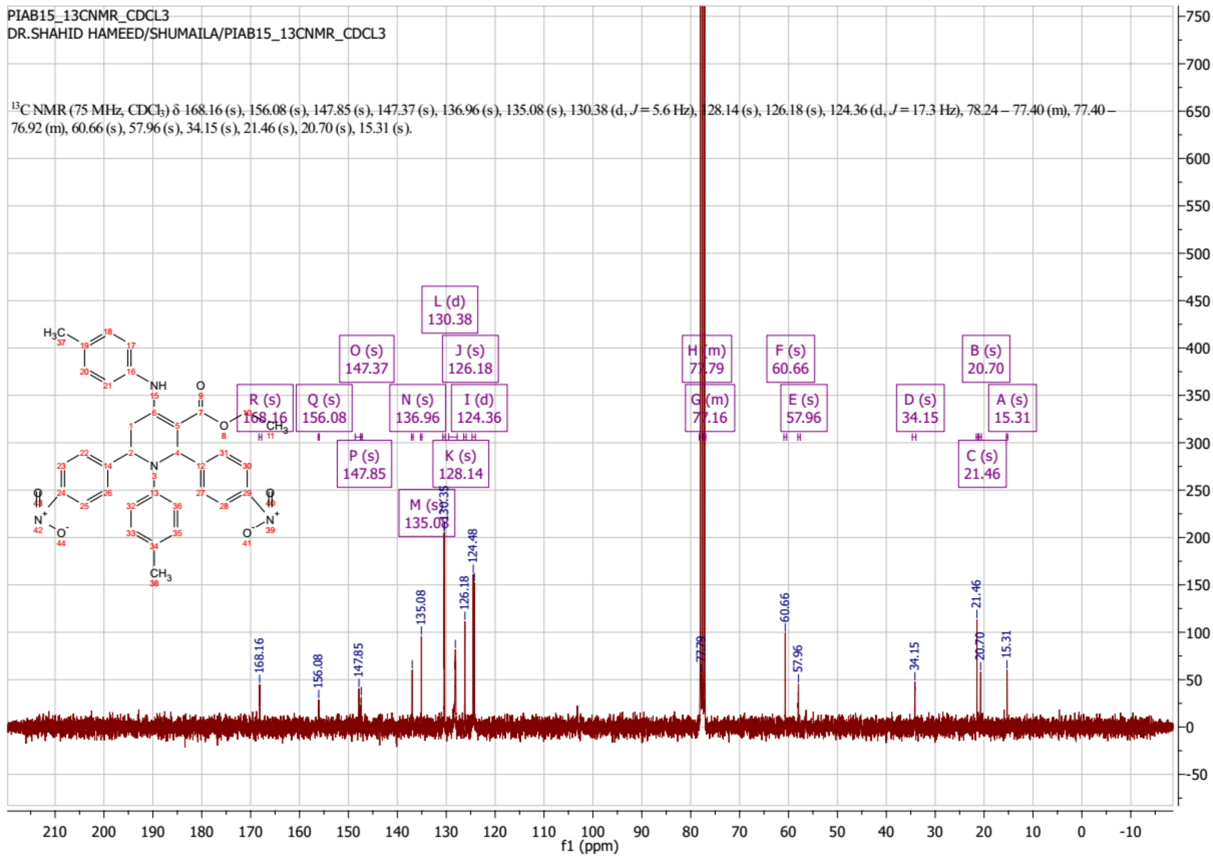

Table S1. The predicted docking scores and the binding features of the synthesized tetrahydropyridines (**4a**–**4o**) against MAO-A and MAO-B enzymes.

Compound Name	MAO-A		MAO-B	
	**Docking Score (kcal/mol)**	**Binding Features**	**Docking Score (kcal/mol)**	**Binding Features**
**4a**	−8.6	ILE207 (2.77Å; π-Sigma); ILE208 (2.25 Å; π-Lone Pair); TYR335 (2.32 Å; π-Lone Pair, 4.66 Å; π-π-Stacked); TRP128 (4.14 Å; π-π-T-shaped)	−7.8	GLN206 (2.48 Å; Hydrogen bond); CYS314 (3.88 Å; π-Sulfur); PHE199 (3.06 Å; π-π-T-shaped); TYR398 (4.18 Å; π-π-Stacked)
**4b**	−7.8	ILE207 (2.97Å; π-Sigma); ILE208 (2.34 Å; π-Lone Pair); TYR335 (2.53 Å; π-Lone Pair, 4.56 Å; π-π-Stacked); TRP128 (4.19 Å; π-π-T-shaped)	−8.3	GLN206 (2.58 Å; Hydrogen bond); CYS314 (3.68 Å; π-Sulfur); PHE199 (3.36 Å; π-π-T-shaped, 3.06 Å; Carbon-hydrogen bond); TYR398 (3.18 Å; π-π-Stacked); AlA101 (3.16 Å; Halogen)
**4c**	−8.8	ILE207 (2.80Å; π-Sigma); ILE208 (2.14 Å; π-Lone Pair); TYR335 (2.41 Å; π-Lone Pair, 4.78 Å; π-π-Stacked, 2.28 Å; π-Sigma); TRP128 (4.19 Å; π-π-T-shaped); PRO113 (2.09 Å; Carbon-hydrogen bond)	−8.3	GLN206 (2.08 Å; Hydrogen bond); CYS314 (4.68 Å; π-Sulfur); PHE199 (4.36 Å; π-π-T-shaped, 4.06 Å; Carbon-hydrogen bond); TYR398 (3.68 Å; π-π-Stacked); AlA101 (3.06 Å; Halogen)
**4d**	−8.8	ILE207 (2.77Å; π-Sigma); ILE208 (2.25 Å; π-Lone Pair); TYR335 (2.32 Å; π-Lone Pair, 4.66Å; π-π-Stacked); TRP128 (4.18 Å; π-π-T-shaped, 2.72Å; π-Sigma); PRO113 (2.05 Å; Carbon-hydrogen bond)	−9.8	GLN206 (2.18 Å; Hydrogen bond); CYS314 (3.98 Å; π-Sulfur); PHE199 (3.36 Å; π-π-T-shaped, 4.06 Å; Carbon-hydrogen bond); TYR398 (3.98 Å; π-π-Stacked); ALA102 (2.59 Å; Carbon-hydrogen bond)
**4e**	−9.3	GLN215 (2.67 Å; Hydrogen Bond, 2.26 Å; Carbon-hydrogen bond); CYS181 (1.97 Å; Hydrogen bond, 2.82 Å; π-Lone pair); TYR444 (2.87 Å; Hydrogen bond, 2.12 Å; π-Donor hydrogen bond); ILE208 (2.25 Å; π-Lone pair); TYR335 (2.25 Å; π-Lone pair, 3.76 Å; π-π-T-shaped)	−9.3	GLN206 (2.46 Å; Hydrogen bond); CYS314 (3.78 Å; π-Sulfur); PHE199 (3.36 Å; π-π-T-shaped, 4.36 Å; Carbon-hydrogen bond); TYR398 (4.00 Å; π-π-Stacked)
**4f**	−10.0	GLN215 (2.07 Å; Hydrogen Bond, 1.91 Å; Carbon-hydrogen bond); TYR444 (1.96 Å; Hydrogen bond, 2.20 Å; π-Donor hydrogen bond); ILE207 (2.46 Å; Carbon hydrogen bond); ILE208 (2.33 Å; π-Lone pair); CYS181 (4.78 Å; π-Sulfur, 2.56 Å; π-Lone pair); LEU173 (2.79 Å; Halogen); PHE177 (4.65 Å; π-π-T-shaped); TYR335 (4.07 Å; π-π-T-shaped)	−6.1	GLN206 (2.00 Å; Hydrogen bond); CYS314 (3.68 Å; π-Sulfur); PHE199 (3.35 Å; π-π-T-shaped, 2.36 Å; Carbon-hydrogen bond); TYR398 (3.99 Å; π-π-Stacked)
**4g**	−8.6	GLN215 (2.17 Å; Hydrogen Bond, 1.74 Å; Carbon-hydrogen bond); ILE207 (2.46 Å; Carbon hydrogen bond); ILE208 (2.72 Å; π-Lone pair); GLY443 (3.26 Å; Halogen); CYS181 (4.87 Å; π-Sulfur, 2.87 Å; π-Lone pair); TYR444 (1.91 Å; Carbon hydrogen bond); LEU173 (2.80 Å; Halogen); PHE177 (4.72 Å; π-π-T-shaped)	−8.2	GLN206 (2.07 Å; Hydrogen bond); CYS314 (3.66 Å; π-Sulfur); PHE199 (3.35 Å; π-π-T-shaped, 2.36 Å; Carbon-hydrogen bond); TYR398 (3.99 Å; π-π-Stacked); TYR435 (2.77 07 Å; Hydrogen bond); AlA101 (3.04 Å; Halogen)
**4h**	−6.3	ILE207 (2.15 Å; Hydrogen bond, 2.61 Å; Carbon hydrogen bond); CYS181 (4.84 Å; π-Sulfur, 2.74 Å; π-Lone pair); LEU173 (2.71 Å; Halogen); ILE208 (2.38 Å; Carbon-hydrogen bond); GLN215 (2.26 Å; Carbon-hydrogen bond); TYR444 (2.05 Å; Carbon hydrogen bond); GLY443 (3.28 Å; Halogen); TYR335 (3.83 Å; π-π-T-shaped)	−7.1	GLN206 (2.17 Å; Hydrogen bond); CYS314 (3.69 Å; π-Sulfur); PHE199 (3.45 Å; π-π-T-shaped, 2.56 Å; Carbon-hydrogen bond); TYR398 (3.79 Å; π-π-Stacked); ALA102 (2.79 Å; Carbon-hydrogen bond); AlA101 (2.04 Å; Halogen)
**4i**	−6.9	ILE207 (2.77 Å; π-Sigma); GLU93 (2.89 Å; Halogen); TRP128 (2.72 Å; π-Sigma, 4.14 Å; π-π-T-shaped); PRO113 (2.05 Å; Carbon-hydrogen bond); ILE208 (2.25 Å; π-Lone pair); TYR335 (2.32 Å; π-Lone pair, 4.66 Å; π-π-Stacked)	−5.5	GLN206 (2.97 Å; Hydrogen bond); CYS314 (3.68 Å; π-Sulfur); PHE199 (3.37 Å; π-π-T-shaped, 3.34 Å; Carbon-hydrogen bond); TYR398 (3.14 Å; π-π-Stacked); AlA101 (3.04 Å; Halogen); ALA102 (3.09 Å; Carbon-hydrogen bond);
**4j**	−7.2	GLN215 (2.67 Å; Hydrogen Bond, 2.26 Å; Carbon-hydrogen bond); CYS181 (1.97 Å; Hydrogen bond, 2.82 Å; π-Lone pair); TYR444 (2.87 Å; Hydrogen bond, 2.12 Å; π-Donor hydrogen bond); ILE208 (2.25 Å; π-Lone pair); TYR335 (2.25 Å; π-Lone pair, 3.76 Å; π-π-T-shaped)	−8.1	GLN206 (2.07 Å; Hydrogen bond); CYS314 (3.66 Å; π-Sulfur); PHE199 (3.57 Å; π-π-T-shaped, 3.33 Å; Carbon-hydrogen bond); TYR398 (3.44 Å; π-π-Stacked)
**4k**	−9.3	CYS181 (1.95 Å; Hydrogen bond, 2.88 Å; π-Lone pair); ILE208 (2.22 Å; π-Lone pair); TYR335 (2.35 Å; π-Lone pair, 3.73 Å; π-π-T-shaped); ILE207 (2.77 Å; Carbon-hydrogen bond); TYR444 (2.08 Å; Carbon-hydrogen bond)	−9.4	GLN206 (2.77 Å; Hydrogen bond); CYS314 (3.70 Å; π-Sulfur); PHE199 (3.55 Å; π-π-T-shaped, 3.42 Å; Carbon-hydrogen bond); TYR398 (3.47 Å; π-π-Stacked)

**4l**	−10.2	CYS181 (2.71 Å; π-Lone pair); ILE207 (2.55 Å; Carbon-hydrogen bond); ILE208 (2.39 Å; π-Lone pair, 2.56 Å; Carbon-hydrogen bond); TYR444 (2.40 Å; Carbon-hydrogen bond, 2.32 Å; π-Sigma; 2.06 Å; π-Donor hydrogen bond); PHE177 (4.66 Å; π-π-T-shaped)	−6.5	GLN206 (2.00 Å; Hydrogen bond); CYS314 (3.69 Å; π-Sulfur); PHE199 (3.57 Å; π-π-T-shaped, 3.35 Å; Carbon-hydrogen bond); TYR398 (3.24 Å; π-π-Stacked); AlA101 (3.14 Å; Halogen)
**4m**	−7.9	PHE177 (2.80 Å; Hydogen bond, 4.66 Å; π-π-T-shaped); CYS181 (2.77 Å; π-Lone pair); ILE208 (2.43 Å; π-Lone pair); ILE207 (2.68 Å; Carbon-hydrogen bond); TYR335 (3.84, 4.47 Å; π-π-T-shaped); TYR444 (2.00 Å; π-Donor hydrogen bond)	−7.2	GLN206 (2.62 Å; Hydrogen bond); ILE171 (2.47 Å; Hydrogen bond); PHE343 (2.61 Å; π-Lone pair); ASN172 (2.98 Å; π-Sigma)
**4n**	−8.1	TYR444 (2.60 Å; π-Sigma); GLY443 (2.65 Å; Carbon-hydrogen bond); ILE207 (2.47 Å; Carbon-hydrogen bond); ILE208 (2.32 Å; π-Lone pair)	−10.3	GLN206 (1.62 Å; Hydrogen bond); TYR188 (2.47 Å; Carbon-hydrogen bond); ASN172 (2.99 Å; π-Sigma)
**4o**	−7.6	CYS181 (2.00 Å; Hydrogen bond, 2.80 Å; π-Lone pair); TYR335 (2.37 Å; π-Lone pair, 3.67 Å; π-π-T-shaped); ILE208 (2.23 Å; π-Lone pair); ILE207 (3.05 Å; Carbon-hydrogen bond); TYR444 (2.00 Å; π-Donor hydrogen bond)	−8.9	GLN206 (2.67 Å; Hydrogen bond); ILE171 (2.77 Å; Hydrogen bond); PHE343 (2561 Å; π-Lone pair); ASN172 (3.04 Å; π-Sigma)

Table S2. IC_{50} graphs of compounds 4a–4o against MAO-A and MAO-B enzymes

CPD	MAO-A	IC_{50}	MAO-B	IC_{50}
**4a**	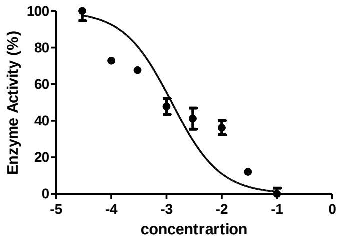	1.29 ± 0.06[a]	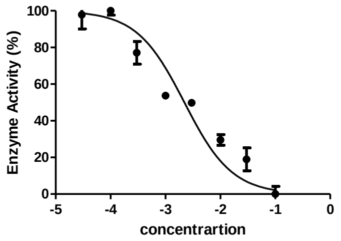	1.70 ± 0.09[a]
**4b**	--	40 %[b]	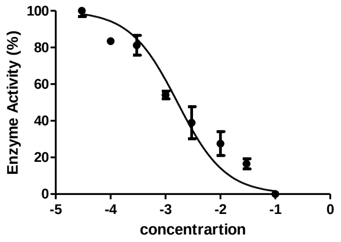	1.54 ± 0.13[a]
**4c**	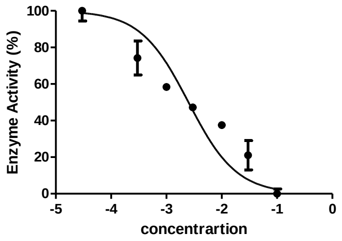	3.44 ± 0.19[a]	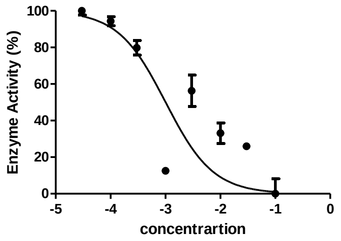	22.3 ± 2.71[a]
**4d**	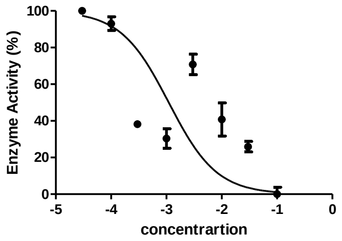	9.13 ± 1.72[a]	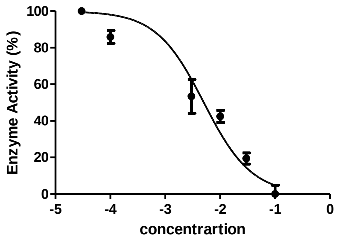	8.89 ± 0.13[a]
**4e**	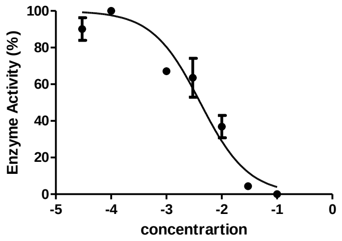	10.7 ± 1.13[a]	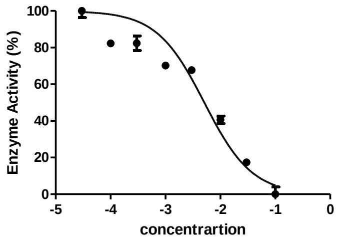	8.03 ± 0.86[a]
**4f**	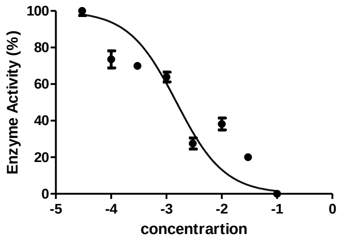	1.26 ± 0.09[a]	----	42%[b]
**4g**	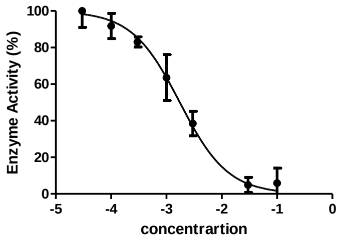	2.03 ± 0.28[a]	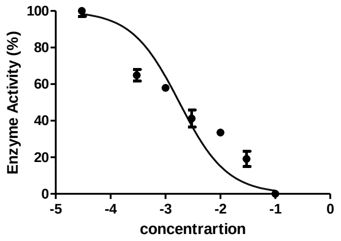	2.07 ±0.01[a]
**4h**	---	31%[b]	---	19%[b]
**4i**	---	46%[b]	---	44%[b]
**4j**	---	33%[b]	---	44%[b]

**4k**	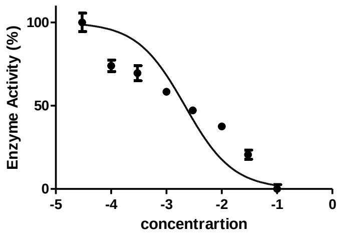	6.01 ± 0.83[a]	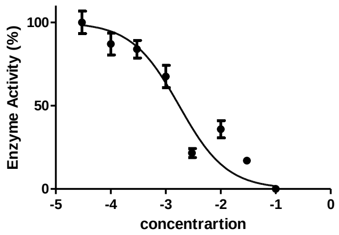	8.47 ± 0.14[a]
**4l**	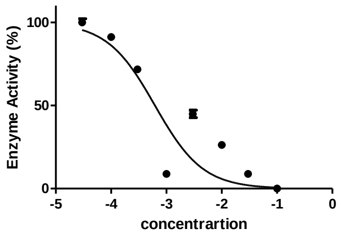	0.40 ± 0.05[a]	---	23%[b]
**4m**	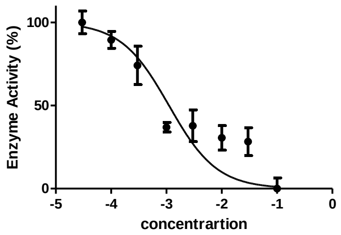	1.19 ± 0.18[a]	---	46%[b]
**4n**	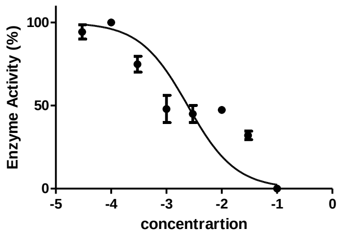	14.4 ± 0.11[a]	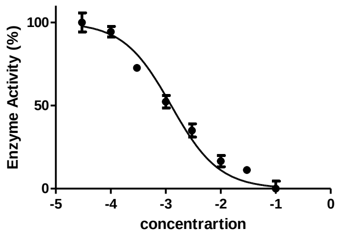	1.01± 0.03[a]
**4o**	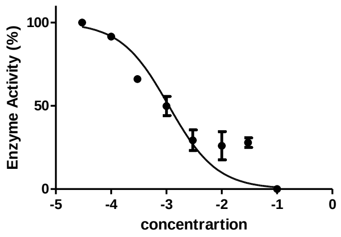	0.59 ± 0.16[a]	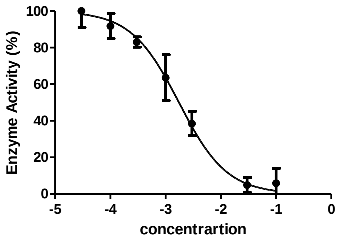	2.67 ± 0.09[a]

[a]All the values are expressed as the mean ± SEM of triplicate determinations.
[b]Percent inhibition observed at 100 µM.
